# Phytochemicals from Plant Foods as Potential Source of Antiviral Agents: An Overview

**DOI:** 10.3390/ph14040381

**Published:** 2021-04-19

**Authors:** Tapan Behl, Gabriele Rocchetti, Swati Chadha, Gokhan Zengin, Simona Bungau, Arun Kumar, Vineet Mehta, Md Sahab Uddin, Gaurav Khullar, Dhruv Setia, Sandeep Arora, Kouadio Ibrahime Sinan, Gunes Ak, Predrag Putnik, Monica Gallo, Domenico Montesano

**Affiliations:** 1 Chitkara College of Pharmacy, Chitkara University, Punjab 140401, India; aaruhichadha1831@gmail.com (S.C.); arundhiman431@gmail.com (A.K.); grvkhullar76@gmail.com (G.K.); dhruvsetia78@gmail.com (D.S.); sandeep.arora@chitkara.edu.in (S.A.); 2 Department for Sustainable Food Process, University Cattolica del Sacro Cuore, 29122 Piacenza, Italy; gabriele.rocchetti@unicatt.it; 3 Department of Biology, Faculty of Science, Selcuk University Campus, Konya 42130, Turkey; gokhanzengin@selcuk.edu.tr (G.Z.); sinankouadio@gmail.com (K.I.S.); akguneselcuk@gmail.com (G.A.); 4 Department of Pharmacy, Faculty of Medicine and Pharmacy, University of Oradea, 410028 Oradea, Romania; sbungau@uoradea.ro; 5 Department of Pharmacology, Government College of Pharmacy, Rohru, Distt. Shimla, Himachal Pradesh 171207, India; vineet.mehta20@gmail.com; 6 Department of Pharmacy, Southeast University, Dhaka 1213, Bangladesh; msu-neuropharma@hotmail.com; 7 Pharmakon Neuroscience Research Network, Dhaka 1207, Bangladesh; 8 Department of Food Technology, University North, 48000 Koprivnica, Croatia; pputnik@alumni.uconn.edu; 9 Department of Molecular Medicine and Medical Biotechnology, University of Naples Federico II, Via Pansini, 5, 80131 Naples, Italy; 10 Department of Pharmacy, University of Naples Federico II, 80131 Naples, Italy

**Keywords:** phytochemicals, plant foods, antiviral agents, viruses, replication, delivery technologies

## Abstract

To date, the leading causes of mortality and morbidity worldwide include viral infections, such as Ebola, influenza virus, acquired immunodeficiency syndrome (AIDS), severe acute respiratory syndrome (SARS) and recently COVID-19 disease, caused by the SARS-CoV-2 virus. Currently, we can count on a narrow range of antiviral drugs, especially older generation ones like ribavirin and interferon which are effective against viruses in vitro but can often be ineffective in patients. In addition to these, we have antiviral agents for the treatment of herpes virus, influenza virus, HIV and hepatitis virus. Recently, drugs used in the past especially against ebolavirus, such as remdesivir and favipiravir, have been considered for the treatment of COVID-19 disease. However, even if these drugs represent important tools against viral diseases, they are certainly not sufficient to defend us from the multitude of viruses present in the environment. This represents a huge problem, especially considering the unprecedented global threat due to the advancement of COVID-19, which represents a potential risk to the health and life of millions of people. The demand, therefore, for new and effective antiviral drugs is very high. This review focuses on three fundamental points: (1) presents the main threats to human health, reviewing the most widespread viral diseases in the world, thus describing the scenario caused by the disease in question each time and evaluating the specific therapeutic remedies currently available. (2) It comprehensively describes main phytochemical classes, in particular from plant foods, with proven antiviral activities, the viruses potentially treated with the described phytochemicals. (3) Consideration of the various applications of drug delivery systems in order to improve the bioavailability of these compounds or extracts. A PRISMA flow diagram was used for the inclusion of the works. Taking into consideration the recent dramatic events caused by COVID-19 pandemic, the cry of alarm that denounces critical need for new antiviral drugs is extremely strong. For these reasons, a continuous systematic exploration of plant foods and their phytochemicals is necessary for the development of new antiviral agents capable of saving lives and improving their well-being.

## 1. Introduction

### 1.1. General Aspects of Viruses

The study of the different existing viruses represents always a hot research topic, as they are considered the leading causes of mortality and morbidity around the world and pose an ongoing threat to public health. This is mainly due to high infectious capacity of many viruses, which is correlated with their specific nature. Viruses are defined as the obligate and intracellular parasites which are comprised of strands of genetic information either in the form of DNA or RNA and are enclosed via lipid core or envelope [1]. The specific feature involves the use of the host cell to propagate and replicate new viruses through the process of acquiring the reproductive tools of the host cell. Hence, this causes cell invasion which leads to ailments such as bloody African fever [2]. Every strain of virus is confined to a unique configuration in respect to surface molecules that work via the same way, i.e., “key-in-a-lock”, which enables and allows entrance of viruses inside host cells [1,2]. The attributed characteristics of viruses include their genetic variation, mode of transmission, replication inside host cells and capability to persist inside the host body. The therapeutic as well as prophylactic measures can be used in order to alleviate and regulate the level of infections caused by viruses. Viruses are not autonomous in nature, hence they necessitate a host body or living cell for the process of replication [1,2,3].

### 1.2. Focus of the Review

This review focuses on three fundamental points: (1) presenting the main threats to human health by reviewing the most widespread viral diseases in the world, thus describing the scenario caused by the disease in question each time and evaluating specific therapeutic remedies currently available. Moreover, (2) it comprehensively describes main phytochemical classes, in particular from plant foods, with proven antiviral activities, the viruses potentially treated with the described phytochemicals. Finally, (3) it considers the various applications of drug delivery systems in order to improve the bioavailability of these compounds or extracts.

The use of natural compounds is taken into account as potential new antiviral remedies representing an alternative and support to conventional drugs in the fight against virus-induced diseases. Specifically, this work is focused on food plants, which in addition to their ability to provide essential nutrients for human nutrition, represent a rich source of biologically active compounds (BACs).

### 1.3. Extraction and Chemical Characterization of Plant Foods and Phytochemicals

Plants and plant foods are known as major sources from which to extract secondary metabolites that can be employed for the synthesis of the same via their metabolic pathway along with the concept of genetic engineering [4]. Currently, epidemiological studies have demonstrated the health promoting potential of phytochemicals from fruits and vegetables, as well as their association with a lower risk for chronic diseases (e.g., cancer and cardiovascular diseases) [5]. One of the major issues in therapeutic use of plants and plant extracts is their standardization, i.e., making methods for their production reproducible in order to always ensure same dosage of bioactive compounds. In this sense, a correct choice of method of extraction and characterization of phytochemicals from vegetable sources, becomes indispensable. In this regard, the impact of new extraction methods to recover high value-added compounds from plant materials, should be considered a focal point for obtaining extracts with high nutraceutical quality [6]. The application of a specific extraction technique must be studied based on the nature of plant matrix to be treated (i.e., leaves, fruit, berries, roots, and tubers). It is always useful to apply techniques that guarantee both quantitative and qualitative recovery of phytochemicals, especially when it is necessary to extract thermolabile or easily oxidizable principles. In this case we should turn to those non-conventional techniques that work by reducing the extraction time and therefore reducing the phenomena of degradation of the phytochemicals [7,8,9]. Likewise, it is of fundamental importance to have a wide range of analytical tools available for the identification and quantification of the phytochemicals present in the plant matrix. The main techniques used for separation of phytochemicals comprises of column chromatography, flash chromatography, thin layer chromatography (TLC), high performance thin layer chromatography (HP-TLC) and high-performance liquid chromatography HPLC, while ultraviolet-visible (UV-Vis) and (infrared) IR spectroscopy, nuclear magnetic resonance (NMR), mass spectrometry (MS), etc. are currently used for identification by means structural elucidation. The well-known bioactive molecule can be identified from numerous chemical libraries and these chemicals undergo screening method for further assurance, while through mass spectrometry, NMR and the study of the spectral characteristics of the molecules it is possible to discover new compounds from different sources.

### 1.4. Biological and Pharmacological Activities of Plant Foods and Phytochemicals

**Figure 1:**
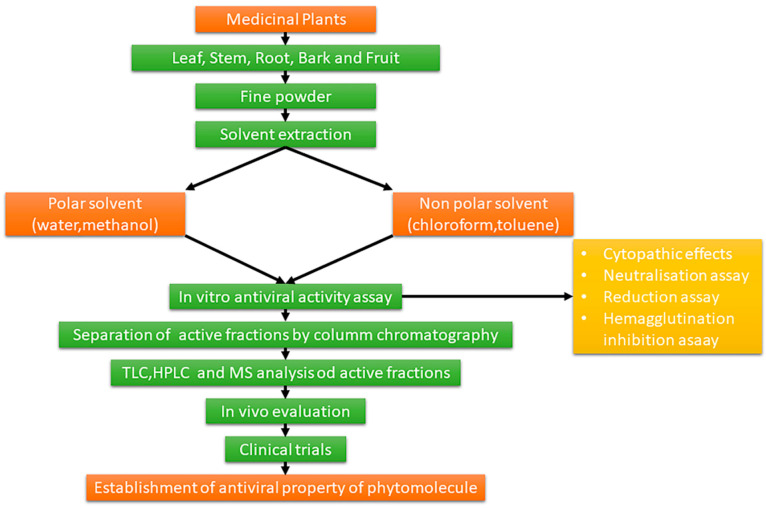
Various processes that a plant has to undergo for the establishment of an appropriate activity for a molecule.

Many investigations have been addressed towards the elucidation of the antiviral activities of compounds from plant world. Once the contribution and quality of the various phytochemicals have been ascertained, their biological activity must be tested using specific assays. The screening of biomolecules for antiviral activity comprises of a number of clinical trials for its teratogenicity, toxicological studies that are carried in vivo and the whole procedure for the same is depicted in Figure 1 [10,11]. This review article describes the numerous chemical classes that are medicinally active such as polyphenols, flavonoids, carotenoids, phytoalexins, minerals, quinines, tannins, lignans, alkaloids, polysaccharides, phytosterols and poly-unsaturated fatty acids that and can be extracted from plants. The phytochemicals presented have important antiviral activities and represent a huge arsenal that can be used against many viral diseases still endemic in various parts of the world. In this context, it must be remembered that Ebola, influenza, acquired immunodeficiency syndrome (AIDS), severe acute respiratory syndrome (SARS), still represent aggressive forms of viral infections. [12]. Influenza alone is responsible for about 250,000-500,000 deaths annually that occurs due to severe diseases. Furthermore, events such as the usage of hypodermic syringes, blood transfusions and organ transplantations lead to the transmission of viral infections from one person to another and leads to increased cases of viral infections [13,14]. The availability of synthetic drugs and vaccines is still too limited to face continuous threats brought by viruses, so turning to the world of natural compounds today represents a logical and intelligent challenge. In this review many examples of antiviral compounds extracted and isolated from plant matrices and in particular from food plants, are reported in order to guarantee a critical contribution for their potential use as new antiviral agents.

## 2. Methodology

### Research Strategy

**Figure 2:**
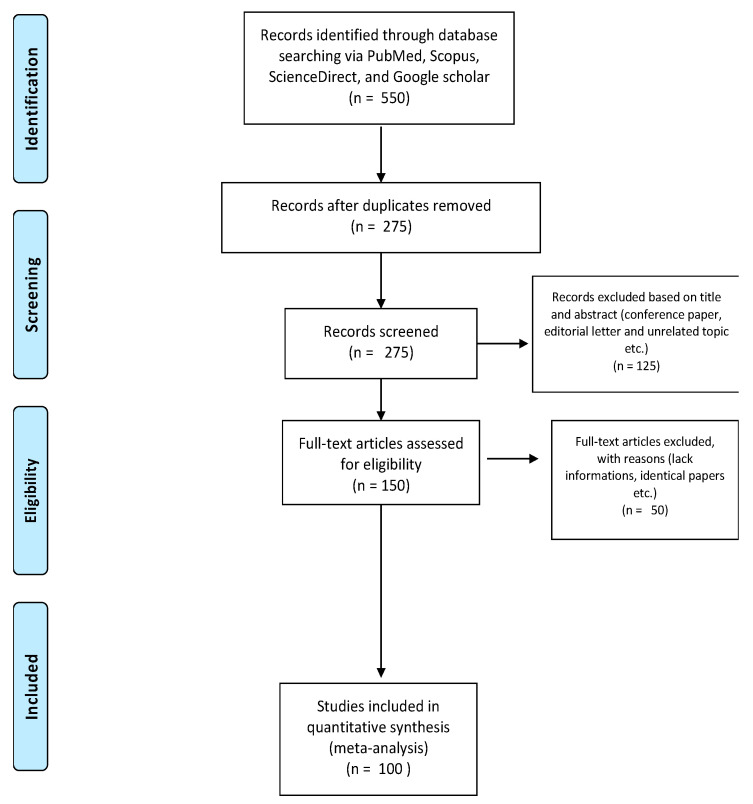
Flow chart of the research strategy adopted.

In order to specifically deepen the most recent literature, papers dated from 2010 to 2020 were screened from key scientific databases (PubMed, Scopus, ScienceDirect, and Google Scholar). Keywords such as virus, antivirals, natural antivirals, phytochemicals, phytochemicals from plant foods, phytochemicals-based remedies, antiviral natural therapy, were searched in order to retrieve the published articles. The papers were restricted to English language. Articles were sorted out and those taken into consideration were chosen firstly by their title and then by scrutinizing their abstract. The research yielded 550 potential papers for designing this review and of which 275 were duplicated records. Moreover, 125 records were excluded for some reasons (conference paper, editorial letter and unrelated topics etc.). The full text of 150 papers were evaluated and 100 papers were included in the review. Figure 2 shows the PRISMA flow chart summarizing the search strategy.

## 3. Viruses

In this section, after a brief introduction on the vicious cycle of viruses, the most important and dangerous viruses for the humans are comprehensively reviewed. For each virus, the classification, the main characteristics, the related-diseases, and any treatments currently available are presented.

### 3.1. Vicious Cycle of Viruses

**Figure 3:**
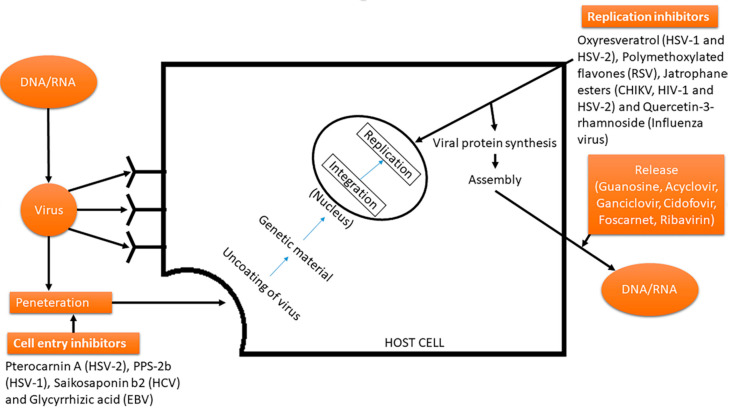
General processes involved in the viral cell in order to replicate itself using host machinery.

The virus attacks via process of adsorption that occurs on a receptor of host cell leading to its penetration followed by uncoating of viral genetic material. The uncoating proceeds and is followed by release of genetic material that further integrates with the genetic composition of the host cell, disrupting and interfering the process of transcription, translation and replication (Figure 3). The control of viral infections is generally implied as a prophylactic measure used to control, regulate and alleviate viral infections from the host [15]. The virus is involved in a typical vicious cycle that includes multiple processes of replication by the host machinery, and along with this, it also includes cellular metabolic pathways that create impediments in the progression of a desired treatment that targets the virion or its process of replication. The treatment should be such that it should not lead to any adverse effects inside host cells or entire body [16,17].

**Figure 4:**
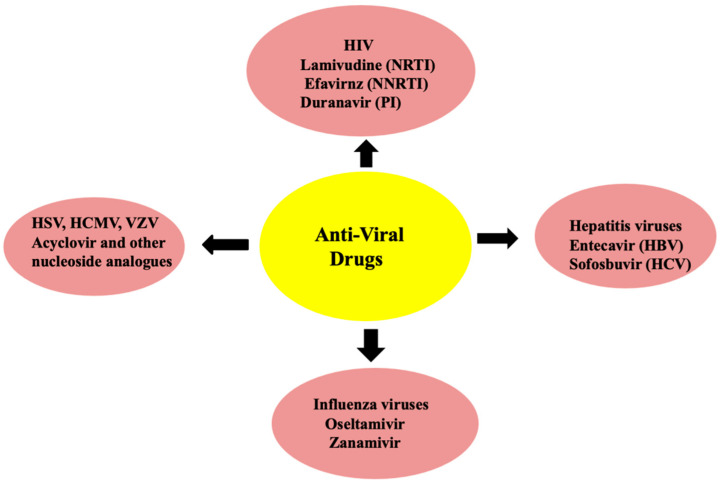
Overview on the main antiviral activities by different classes of drugs. (HSV—virus herpex simplex; HMCV—human cytomegalovirus; VZV—varicella zoster virus)

Viral enzymes generally play a distinct and major role in triggering and developing associated diseases. Hence, effective approaches for such treatment can be through the inhibition of these viral enzymes that ultimately leads to stopping of the viral replication. For instance, the acycloguanosine, based on the nucleosides isolated from a Caribbean sponge (*Cryptotethya crypta*) is able to provide a treatment against herpes infection. The mechanism of action interrupts the processing of viral enzymes that are responsible for the synthesis of nucleotide and its analogous. The maturation of virus may be carried out via proteinase; hence this enzyme is a good target for the inhibition via a potential mechanism [18,19]. Figure 4 schematically reports the most important anti-viral drugs against human immunodeficiency virus (HIV), hepatitis virus, influenza viruses, and others.

### 3.2. Respiratory Syncytial Virus

Every year millions of people fall ill with respiratory syncitial virus (RSV) and there are more than 100,000 deaths. Although everyone can contract RSV, young and old are the hardest hit. In 2017, World Health Organization (WHO) estimated that this virus causes approximately 33 million serious respiratory infections each year, three million hospitalizations and nearly 60,000 deaths of children under the age of 5. RSV was initially originated from colony of chimpanzees that includes coryza agents. RSV not only affects children, but also adults. Regarding the annual incidence of RSV, a trend similar to influenza A has been observed in adults in fact, currently, healthy adults that get RSV present specific symptoms such as cough, rhinorrhea, and congestion [20]. The infection of RSV is constricted to the upper respiratory tract that involves nasopharynx with incubation period of 3–5 days. The hyperinflation of distal airways is mainly due to necrosis in epithelial cells that leads to intra-luminal plugs of mucous, which forms a ball valve type obstruction of airway [21]. The prominent role is played by cellular and individual immunity with the decrease immunodeficiency. The clinical manifestations involve characteristic symptoms such as fever, cough and rhinitis. Other clinical manifestations of this disease include bronchitis, pneumonia, and croup. Along with bronchitis, air trapping is observed in some cases, while two thirds of cases are accompanied by bronchiolitis and pneumonia [22]. Although several research teams are working hard, no vaccine was found and approved to prevent this infection. Actually, it is possible to use Palivizumab as a prophylaxis against RSV in high-risk infants, but other pharmacological treatments are not available beyond this [23]. A real strategy can be the administration of antibodies in infants that helps with the prevention of RSV virus [24].

### 3.3. Para-Influenza and Influenza Virus

Para-influenza belongs to species of genus Paramyxovirus from family of Paramyxoviridae which are commonly called human parainfluenza viruses (HPIVs). This is a group of viruses that causes upper and lower respiratory illnesses and closely resembles common cold. Para-influenza viruses differ from RSV as it can re-infect the host and this event can lead to infections of upper respiratory tract [25]. The parainfluenza includes Parainfluenza virus type 1 (or Para 1) that causes laryngotracheobronchitis in infants and children. Further, Para influenza type 2 whose clinical manifestations resemble those of Para 1, but with less danger for hosts as compared to the type 1. Para influenza virus type 3 can lead to pneumonia, as well as bronchiolitis, with major adverse effects inflicting to infants below six months of age. Para influenza virus type 4 is rarer than the other types and clinical manifestations are not detected easily [26].

The Orthomyxoviridae family includes several influenza A virus subtypes that originally resided only in some bird species. Various influenza A virus subtypes were identified between 2003 and 2004, such as H7N7 in Netherlands, H9N2 in Hong Kong and H7N2 in the United States, H5N1 in Southeast Asia, H7N3 in Canada and H10N7 in Egypt [27]. In particular, case of avian influenza virus, caused by subtype H5N1, which occurred in 1997 in Southeast Asia, is today remembered as a very pernicious and highly pathogenic epidemic. This virus caused extensive poultry epidemics but even more serious as it led to death of many people because it was transmitted from bird to man. At that time the scientific world wondered if a pandemic risk was taking place [28,29]. Currently, there is no vaccine to protect against infection caused by human parainfluenza viruses. There is no specific antiviral treatment for HPIV illness. Treatment of parainfluenza virus infection is symptomatic, to relieve symptoms acetaminophen and ibuprofen could be useful and other over-the-counter medications for pain and fever.

### 3.4. Coronaviruses

The term coronavirus refers to a group of large, enveloped, positive-stranded RNA viruses belonging to *Coronavirus* genus. These viruses usually cause mild to moderate upper respiratory tract diseases, such as common cold but also other diseases (mainly to gastrointestinal and central nervous system) in both humans and animals [30]. The coronavirus family includes several hundred viruses that are widespread among some types of animals including bats, pigs and cats [31]. The transmission of these viruses to humans is not a very widespread event, however sometimes a spillover event occurs-with the passage of virus to humans and related development of diseases [32]. Typically, when this transmission to humans occurred, only mild to moderate diseases developed. However, in the past twenty years new coronaviruses have been responsible for three dramatic events, causing serious widespread disease and death.

An infectious agent belonging to coronavirus genus, severe acute respiratory syndrome-associated coronavirus (SARS-CoV) [33], caused more than 750 deaths distributed in 26 countries in late 2002 and the first half of 2003. In total there were more than 8000 cases of people infected with this virus, with a fatality rate of 9.6%. People who contracted SARS had symptoms such as fever, cough and dyspnoea, while common serological tests recorded lymphopenia, thrombocytopenia and C-reactive protein [34]. From a histopathological point of view, patients with full-blown SARS disease, have also reported serious problems in lungs, recording significant alveolar damage, which led the patient to develop pneumonia [35]. Some studies showed that this virus could originate from animals, by means of an interspecies transmission [36,37]

The most accepted hypothesis on how the virus was transmitted, was through droplets and by direct contact, therefore all precautions to avoid contagion through droplets and contact were used at that time in the prevention of nosocomial transmission of SARS [38].

A second coronavirus outbreak occurred in March 2012. A beta-coronavirus was responsible for a dramatic pathological condition that included severe respiratory distress with high mortality [39,40]. Many countries were affected by this virus, the most affected were those of the Middle East such as Egypt, Iran, Jordan, Kuwait, United Arab Emirates, Yemen, and for this reason the virus was called Middle East respiratory syndrome coronavirus (MERS-CoV). The spread of this virus, however, soon crossed borders of Middle East and also infected European countries such as Austria, France, Germany, Greece, Italy, and finally some outbreaks also developed in the United States. After about three years, 1075 people infected with MERS-CoV were registered, as well as 404 deaths related to this virus. As documented, taking into account all the emergencies it caused, MERS-CoV has infected 2,494 people in 27 countries and overall resulted in death of at least 858 people until November 2019 [41]. As previously for SARS, also in this case the origin of the virus was mainly from animals and in this case bats and camels [42]. To date, there are no antivirals or vaccines approved by FDA for treatment and prevention of MERS-CoV infection. The only possible treatment is constituted by supportive therapy and above all preventing complications in respiratory level. All of these highlights dramatic need for direct-acting antiviral drugs [43,44]. The latest case of coronavirus, the most serious case, has occurred since late 2019. The International Committee on Taxonomy of Viruses (ICTV) named this novel virus as SARS-CoV-2, infectious agent of coronavirus disease (COVID-19). In chronological order, this represents the third largest coronavirus outbreak of the past 20 years, but it is certainly first from the point of view of socio-economic and health impacts. Its diffusion has been remarkable, involving more than 200 countries in the world, for this reason has been declared a global pandemic by the World Health Organization. In late 2019, WHO China Country Office became aware of pneumonia cases of unknown etiology in Wuhan, Hubei. In early January 2020, Chinese national authorities reported a total of 44 patients with pneumonia of unknown etiology to WHO (WHO, 2020). After these first cases in province of Hubei virus spread throughout China causing thousands of deaths and shortly afterwards the virus reached Italy and from here the other European countries. At the time of authoring (2 June 2020), there are 6,194,533 cases confirmed worldwide since the outbreak and 376,320 deaths [45]. During this epidemic, infected people presented symptoms such as fever, breathing problems, cough, severe acute respiratory syndrome and severe kidney failure [46].

Once again, this virus appears to derive from animal species that are normally able to host it. According to some scientists, at the end of 2019, there would have been a spike mutation, and this would have caused the transmission of the virus to humans [47]. The first management strategy to fight COVID-19 outbreak was mainly based on supportive therapy and treatment of symptoms, in an attempt to prevent respiratory failure [48,49]. Then, several world research-programs have evaluated different therapeutic solutions to counteract the advance of the virus. In particular, remdesivir was proposed as a promising candidate for treating COVID-19. Remdesivir is a nucleotide analogue prodrug that has shown antiviral efficacy against SARS-CoV, MERS-CoV and SARS-CoV-2 in both cellular and mouse models [50]. Favipiravir was another antiviral drug involved in clinical trials to evaluate its effectiveness against COVID-2019 [51]. Favipiravir is a purine nucleic acid analogue and potent RNA-dependent RNA-polymerase inhibitor (RdRp) approved for use in influenza and is also considered in numerous clinical studies. Favipiravir has been considered for compassionate use in COVID-19 by taking advantage of its mechanism of action that inhibits the RdRp virus and relying on safety data in previous clinical studies. However, the exact efficacy of favipiravir has been not yet confirmed by ad-hoc clinical studies [51]. Since the end of 2019, many clinical data have reported mild or severe cytokine storms in most severe patients, and these were the main causes of death. In severe cases there was a sustained reduction in the percentage of lymphocytes compared to mild cases. Furthermore, in severe COVID-19 cases a decrease in CD8 + T cells was reported, while inflammatory cytokines (IL-6, IL-10, IL-2 and interferon-gamma (IFNγ)) increased in peripheral blood. In light of this, cytokine storm treatment has become the target to consider for saving serious patients with COVID-19. As known, interleukin-6 (IL-6) has a key role in cytokine release syndrome. Starting from this assumption, it is possible to imagine a therapy focused on blocking IL-6 signal transduction pathway. This represents the scientific basis justifying the use of drugs capable of blocking the IL-6 receptor (IL-6R) such as Tocilizumab [52,53,54]. At the time of this review (March 2021) there are now several vaccines that are in use. In particular, scientist around the world have worked for designing different types of vaccines, based on different mechanisms of action including a) Inactivated or weakened virus vaccines, which use a form of the virus that has been inactivated or weakened so it doesn’t cause disease, but still generates an immune response; (2) Protein-based vaccines, which use harmless fragments of proteins or protein shells that mimic the COVID-19 virus to safely generate an immune response; (3) Viral vector vaccines, which use a safe virus that cannot cause disease but serves as a platform to produce coronavirus proteins to generate an immune response; 4) RNA and DNA vaccines, a cutting-edge approach that uses genetically engineered RNA or DNA to generate a protein that itself safely prompts an immune response. According to WHO progress and regulatory document about vaccines (16 February 2021), three vaccines present a complete and finalized assessment about their administration to humans, namely BNT162b2/COMIRNATY Tozinameran (INN) (i.e., a nucleoside modified mRNA, from Pfizer (New York, NY, USA)/Biontech (Mainz, Germany)), AZD1222 (i.e., a recombinant ChAdOx1 adenoviral vector encoding the Spike protein antigen of the SARS-CoV-2, from AstraZeneca (Cambrige, UK) /SK Bioscience (Pangyo-ro Bundang-gu Seongnam-S, Korea), and Covishield ChAdOx1_nCoV19 (i.e., a recombinant ChAdOx1 adenoviral vector encoding the Spike protein antigen of the SARS-CoV-2, from Serum Institute of India, (Maharashtra, India).

### 3.5. Herpes

Herpes virus belongs to the family of viruses that are most dangerous pathogens for humans and comprises of HSV-1 and HSV-2. HSV-1 can cause encephalitis and orofacial infections while HSV-2 infections cause injury and inflammation around genital area and is sexually transmitted. Lesions and infection in sensory neurons are inflicted in both of these cases, while the extent of pathogenicity is based on increased lesions and latency. The herpes simplex virus (HSV) is composed of about 84 different polypeptides and each single protein can encoded in numerous ways and perform various functions. The infection is initiated as soon as HSV attaches to surface of cell receptor and fuses with plasma membrane in order to release viral DNA inside the nucleus [55,56]. The replication of HSV involves three rounds of transcription. HSV has the ability to cause infections including neuroinvasiveness, neurotoxicity and latency period during which it invades inside brain, destroys the neurons of brain, and then settles to a dormant state. Majority of HSV infections are mainly caused by HSV-2 during sexual transmission, as well as from mother to fetus transfer. The symptoms appear after a time period of 2–12 days. The different types of lesions in symptomatic oropharyngeal disease include intraoral lesion during primary infection, followed by recurrent orolabial lesions that may present with increased pain, itching and swelling of the lips. The other skin infections related to HSV-1 include eczema herpeticum and atopic dermatitis. The HSV-2 is responsible for genital herpes that generally is characterized by macules and papules subsequently turn to vesicles, ulcers and pustules. The lesions in vesicle genital area generally last for 8–10 days [57]. Actually, there are no effective vaccines or prophylaxis providing a complete protection or immunity from virus, which is endemic worldwide. As regards pharmacological treatment of HSV-2, antivirals are currently used to control viral replication. The most commonly used drugs are Acyclovir, its analogue Valacyclovir and Famcyclovir (prodrug of Pencyclovir). Their mechanism of action, as nucleoside analogues, is to specifically inhibit the DNA polymerase of herpesvirus [58]. We can conclude that, to date, first-choice drugs used for the treatment of HSV-1 and HSV-2 are still represented by second generation of antivirals based on acyclic nucleosides such as aceaciclovir, valacyclovir, ganciclovir, penciclovir, and famciclovir.

### 3.6. Rotavirus

Rotavirus belongs to family of Reoviridae and comprises of double stranded RNA which can be separated on the basis of gel electrophoresis. They include six structural proteins that are combined in order to form triple layers. Rotaviruses comprise nine groups (A-I) as established by ICTV (available at https://talk.ictvonline.org/taxonomy/, accessed on 28 March 2021, which are based on presence of antigens and their subtypes [59]. The rotavirus from group A, B and C causes to infection in humans. Rotaviruses are most durable and their survival time in recreational and potable waters can persist for weeks. Currently, rotavirus infections are a major cause of severe and dehydrating gastroenteritis in children. In particular, rotavirus type A (RVA) is pathogenic form that most commonly causes pathology in its acute form in humans, causing approximately 215,000 deaths of children under the age of five each year [59,60,61]. Considering only Europe, RVA resulted in 75,000–150,000 hospitalizations of children presenting with symptoms of acute gastroenteritis [62]. The severe form of diarrhea that occurs as a consequence of rotavirus infection occurs because this agent mainly infects enterocytes leading to destruction and therefore causing malabsorption. In addition to diarrhea, rotavirus infection can induce vomiting, malaise and fever [63]. Vomiting is considered aggravating because it contributes to patient’s dehydration. Patients who experience vomiting and diarrhea at the same time have greater difficulty recovering as oral rehydration therapy is less effective and other therapeutic regimens are generally less successful. [64]. To date, vaccination is one of the most effective strategy for fighting RVA. Already since 2006, oral rotavirus vaccines have been authorized and are used in more than 100 countries around the world [65]. The vaccination policy has given its benefits, in fact without this important weapon, gastroenteritis associated with rotavirus has caused over 500,000 deaths in children under the age of 5 [66]. Moreover, for RVA it has been shown that animals can represent the first cause of infection for humans. In fact, both humans and animals have the same RVA genotypes. Although animal derived RVA appears to be unable to infect or spread efficiently in the new host, however, the danger is always very high when considering the likelihood that these strains will acquire genetic segments of human origin. If such a hypothesis were to occur, the contagion would occur in a very effective way with sure development of the pathology [67].

### 3.7. Dengue Virus

Dengue virus, also referred to as DENV, belongs to the family of *Fliviviridae* and can be transmitted to humans by mosquito bite. Currently, dengue still poses a serious threat to public health. In 2015, more than two million cases of dengue occurred in America alone, and about ten thousand infected people had serious clinical symptoms often due to hemorrhagic complications. It is estimated that this disease is endemic in over 100 countries in Southeast Asia, the Americas, the western Pacific, Africa and as far as the Eastern Mediterranean regions [68]. Todays, dengue, is one of the most important arboviruses capable of affecting humans and is widespread mainly in subtropical and tropical countries, with environmental playing an important role in the development and proliferation of *Aedes aegypti* and *Aedes albopictus* mosquitoes, i.e., main vectors transmitting the virus to humans. They are classified into four major subtypes namely DENV-1, DENV-2, DENV-3 and DENV-4 [69]. The initial infection of dengue virus is characterized by mild flu-like symptoms that subsequently can change to dengue fever. This can lead to development of the most hazardous form of dengue hemorrhagic fever (DHF) which is characterized by increased vascular fragility and coagulopathy. In severe cases, latter step can progress to dengue shock syndrome (DSS) and possibly cause death. The onset of disease is characterized by fever that is followed by headache, myalgia, gastrointestinal discomfort, rashes and hemorrhagic manifestations [70]. Currently, there are no known specific treatments for dengue, and no document approving new drugs against this disease has been produced in recent times by official validation bodies such as the US Food and Drug Administration (FDA). The only therapeutic weapon consists of adequate and timely medical assistance with supportive therapies. People who heal, develop immunity for life, but only for the infectious serotype while only partial and transitory immunity develops to other serotypes. There have been several problems in developing vaccines that are able to immunize patients against DENV and, to date, various techniques have been used to develop vaccines potentially usable for this purpose. Some therapeutic agents have been considered for the treatment of dengue, primarily chloroquine [71]. This drug was found to inhibit the replication of the virus in vitro but does not have a great impact on the duration of the viral infection, in addition to presenting various side effects [72]. Another drug tried to treat dengue was balapiravir, an inhibitor of hepatitis C virus replication in vivo. Although it appears to be well tolerated, balapiravir did not give very positive results, in fact it was not able to reduce plasma concentrations of cytokines or to ensure the time to eliminate the fever [73].

### 3.8. Human Immunodeficiency Virus

The other virus that can have an adverse effect on humans include human immunodeficiency virus (HIV) that causes acquired immunodeficiency syndrome (AIDS) and major worldwide epidemic for which vaccine is still not found. The human immunodeficiency viruses 1 and 2 (HIV-1, HIV-2) directly derived from simian immunodeficiency viruses (SIVs) of primates, thus both having a zoonotic origin. HIV-2 has a more limited geographical spread and appears to be less pathogenic than HIV-1, while HIV-1 is responsible for worldwide HIV pandemic [74]. HIV is an RNA virus that belongs to Lentivirus genus, of the Retroviridae family [75]. This latter is a particular viral family, that of retroviruses, with an absolutely unique replication mechanism. By means of a specific enzyme, reverse transcriptase, retroviruses are able to transform their RNA gene pool into a double-stranded DNA. This goes into the DNA of the infected cell (called “host cell” or “target cell”) and from there directs the production of new viral particles [76]. Retroviruses characterized various vertebrate species, capable to cause various diseases in humans and animals. Retroviruses have often been linked to various autoimmune diseases that affect the human body at different levels, inducing sometimes serious pathologies ranging from various anemias to CNS diseases and immunodeficiency syndromes [77]. The estimates of spread of this virus are truly dramatic. At the end of 2017, the number of people infected was close to 37 million [78]. However, in addition to the growing concern for considerable number of cases, what is even more worrying is high degree of correlation between AIDS and some serious forms of cancer, primarily non-Hodgkin’s lymphoma [79]. In fact, these cancerous forms are considered as the first evident stage of clinically relevant immunosuppression. In fact, in the five-year period 1991–1995 it was calculated that in the United States people infected with HIV presented 2800-fold elevated risks for KS, 10-fold for NHL and three-fold for cervical cancer respect to the general population [80]. The course of HIV disease typically proceeds with fever and lymphadenopathy, symptoms that disappear for 2–15 years following seroconversion. During this period, viral replication continues at a very high rate every day causing the formation of a large number of infected lymphocytes, which are replaced almost equally quickly. This rapid turnover of HIV and its enormous diversity underlie the problems in producing long-term effective antiretroviral drugs together with the development of an effective HIV vaccine [81]. Available treatments that improved the lifespan of the individuals suffering from HIV comprises of antiretroviral treatment (ART). Instead, highly active antiretroviral therapy (HAART) is insufficient to eradicate the HIV, but it is mainly responsible for the suppression of viral load [82]. ART has the great merit of controlling HIV infection, but this does not mean that it permanently cures the disease. Infected people must continue taking ART indefinitely, turning HIV infection into a chronic disease. HAART was developed about 20 years ago and consisted of a useful combination of antiviral agents. This therapeutic scheme uses a number of drugs with specific inhibitory effects on HIV replication. Specifically, these antiviral agents belong to six distinct classes of drugs with different mechanisms capable of inhibiting HIV replication at different stages of the HIV life cycle [83]. The advantage of this treatment has proven to be remarkable, prolonging the survival of HIV/AIDS patients by about 7–10 years compared to the results obtained from the use of a single drug [84]. Although this therapy has clearly contributed to improving the condition of infected people, recent studies showed that exposure to antiretroviral drugs may have marked side effects, regardless of HIV status [85]. Different plants and phytochemicals exerting activity against numerous viruses are presented in Table 1.

**Table 1:** Different plants and phytochemicals exerting activity against numerous viruses.

Plant	Family	Virus	Types of Extract	Antiviral Compounds	Ref.
*Aegle marmelos*	Rutaceae	Human coxsackie viruses B1-B6, nuclear polyhedrosis virus	Hexane, ethyl acetate and methanol and aqueous	Marmelide, seselin	[86] [87]
*Allium cepa* L.	Amaryllidaceae	SARS-COV, Newcastle disease virus	Ethanol	Quercetin, allicin, thiosulfinates	[88] [89]
*Allium sativum* L.	Amaryllidaceae	DENV, common cold virus, influenza virus A and B, HIV, HSV-1, HSV-2, Newcastle Disease Virus	Aqueous	Quercetin, allicin, thiosulfinates, ajoene	[90] [91] [89] [92]
*Aloe vera*	Liliaceae	HSV-1, HSV-2;	Gel	Unknown	[93] [94]
*Artocarpus integrifolia*	Moraceae	(SA-11) and human (HCR3) rotaviruses, HIV	-	Jacalin	[95]
*Balanites aegyptiaca*	Zygophyllaceae	VSV T2	Hexane, 80% methanol and water	Unknown	[96]
*Berberis vulgaris* L.	Berberidaceae	Enterovirus 71, human cytomegalovirus (HCMV), CHIKV	-	Berbamine, berberine	[96] [97] [98]
*Camellia sinensis*	Theaceae	HBV, HCV, HSV-1, HIV-1, caliciviruses	-	Catechins, quercetin, gallic acid, theaflavins, theaflavin-3,3′-digallate, theaflavin digallate, Epigallocathechin-3-gallate, (-)-epicatechin gallate	[99] [100] [101] [102,103]
*Capparis spinosa*	Capparidaceae	HSV-2 HSV-1	Methanol	Unknown Protein	[103] [104]
*Carica papaya* L.	Caricaceae	DENV	Aqueous	Unknown	[105] [106] [107] [108]
*Cassine xylocarpa*	Celastraceae	HIV-1	-	Pentacyclic lupane-type triterpenoids	[109] [110]
*Cistus incanus*	Cistaceae	HIV-1, HIV-2	-		
*Citrus aurantium L.*	Rutaceae	DENV; HIV-1, HSV types 1 and 2, influenza, and yellow fever.	-	Polysaccharides, polyphenols	[111] [112] [112]
*Curcuma longa*	Zingiberaceae	HSV types 1 and 2	-	Curcumin	[113] [114]
*Diospyros kaki*	Ebenacee	Influenza virus H3N2, H5N3, HSV-1, vesicular stomatitis virus, Sendai virus, poliovirus, coxsachievirus, adenovirus, rotavirus, feline calicivirus, mouse norovirus, Newcastle disease virus	Aqueous	Licocoumarone, licoflavonol, glyasperin D, luteolin, vitexin, apigenin-7-O-glucoside; tannins	[115] [116]
*Euphorbia hirta*	Euforbiacee	HIV-1, HIV-2, SIV mac 251	Aqueous	Flavonoids	[117]
*Euphorbia spinidens*	Euforbiacee	HSV type 1	Methanol	Unknown	[118]
*Ficus carica*	Moraceae	HSV-1 HSV-1, ECV-11 and ADV influenza virus	Latex	Unknown	[119]
*Glycyrrhiza glabra*	Fabaceae	HCV, HSV, CVB3, DHV, H5N1, Influenza virus, HRSV,	Aqueous, methanolic and ethanolic	Glycyrrhizin, 18 β-glycyrrhetinic acid, liquiritigenin, licochalcone A and E, glabridin	[120] [121]
**Glycyrrhiza uralensis**	Fabaceae	HCV; Rotavirus diarrhea	Methanol	Glycycoumarin, glycyrin, glycyrol, liquiritigenin, isoliquiritigenin, licochalcone A and glabridin	[122] [123]
*Hyssopus officinalis*	Lamiaceae	HSV type 1 and 2	-	Unknown	[124]
*Lycium barbarum*	Solanaceae	NDVs	-	Polysaccharides	[125] [126]
*Melissa officinalis*	Lamiaceae	HSV-1, HSV-2 HIV, influenza virus	Aqueous	Essential oils	[127]
*Mentha pulegium*	Lamiaceae	HSV type 1	Methanol	Unknown	[128]
*Moringa peregrina*	Moringaceae	HSV type 1	Aqueous	Unknown	[129]
*Moringa oleifera*	Moringaceae	HIV, HSV, HBV, EBV, FMDV and NDV.	-	Flavonoids and phenolic acids	[130] [131]
*Myristica fragrans*	Myristicaceae	Human rotavirus		Unknown	[132]
*Olea oleuropaea*	Oleaceae	NDV	Aqueous	Unknown	[133]
*Panax ginseng*	Araliaceae	RSV, influenza virus, HIV, HSV, hepatitis virus, enterovirus, coxsackievirus, norovirus, rotavirus rhinovirus,	-	Epigallocatechin gallate, theaflavin digallate, genistein, hesperidin, neohesperidin, diosmin, pectic polysaccharides; ginsenosides	[132,134]
*Panax notoginseng*	Araliaceae	Influenza A virus	Aqueous	Unknown	[135]
*Phyllanthus acidus*	Phyllanthaceae	HBV	-	Highly oxygenated norbisabolane sesquiterpenoids, phyllanthacidoid acid methyl ester	[136]
*Phyllanthus emblica*	Phyllanthaceae	Influenza A virus strain H3N2 HBV	-	Highly oxygenated norbisabolane sesquiterpenoids Sesquiterpenoid glycoside dimers	[136,137]
*Piper longum*	Piperaceae	HBV	-	Longumosides A and B, and two amide alkaloids	[138]
*Prunella vulgaris*	Lamiaceae	HIV-1 Ebola virus	Aqueous	Unknown	[139]
*Psidium guajava*	Myrtaceae	H1N1 viruses	Aqueous	Tannins and polyphenols	[140] [141]
*Quercus persica*	Fagaceae	HSV-1	Aqueous	Unknown	[142]
*Salacia reticulata*	Celastraceae	H1N1 viruses	Aqueous	Unknown	[143]
*Sanguisorba minor*	Rosaceae	VSV, HSV-1 HIV	Methanol/Water	Unknown	[144] [145]
*Solanum nigrum*	Solanaceae	HCV	Methanol	Unknown	[146]
*Spondias lutea*	Anacardiaceae	Human rotavirus, HSV type 1	-	Hydrolysable tannins, O-glycosylated flavonoids, phenolic acids, and a carbohydrate	[147]
*Taraxacum officinale*	Asteraceae	HCV Influenza virus type A, H1N1	Methanol	Unknown	[148]
*Thymus vulgaris*	Lamiaceae	HIV-1	Methanol	Unknown	[149]
*Thymus carmanicus*	Lamiaceae	HIV-1	Aqueous	Unknown	[150]
*Thymus daenensis*	Lamiaceae	HIV-1	Methanol	Unknown	[149]
*Thymus kotschyanus*	Lamiaceae	HIV-1	Aqueous	Unknown	[151]
*Viola diffusa*	Violaceae	HBV	-	Friedelolactones	[152]
*Zataria multiflora*	Labiate	HSV-1	-	Rosmarinic acid	[153]
*Zingiber officinale*	Zingiberaceae	Chikungunya virus (CHIKV)	Aqueous	Unknown	[154]

Drug delivery systems employed for the treatment of viral infections are summarized in Table 2.

**Table 2:** Drug delivery systems employed for the treatment of viral infections.

Phytochemicals or Extracts	Potential Antiviral Activity	Delivery System Strategy	Reference
Myricetin	HIV, RLV, influenza	Self-nanoemulsifying drug delivery systems SNEDDS, nanogel, mixed micelles, cocrystal, nanoencapsulation, nanosuspension	[155]
Apigenin	Influenza A, HCV, Enterovirus 71, FMDV, ASFV.	Solid dispersion, W/O/Wemulsion, O/W microemulsion, mixed micelles, phospholipid phytosome, self-microemulsifying drug delivery systems (SMEDDS) pellets	[156,157,158]
Baicalin	DENV, RSV, HIV, Hepatitis B virus (HBV), influenza virus, NDV, enterovirus 71.	Liposome, mixed micelles, polymeric micelles, SNEDDS, SMEDDS, nanoparticles, nanocrystals, inclusion complex solid dispersion	[159,160]
Catechins	HBV, HSV, EBV, Adenovirus, HIV, HCV, Influenza virus, DENV, JEV, TBEV Zika Virus (ZIKV), CHIKV, HTLV-1, Rotavirus, Enterovirus EV71, EBOV, PRRSV, VHSV, IHNV, SVCV, GCRV.	Microparticles, calcium pectinate gel particles, chitosan nanoparticles, Nanoparticles of Polylactic Acid–polyethylene Glycol, poly(lactic-co-glycolic acid) nanoparticles, gold nanoparticles, colloidal complexes, liposomes, nanoemulsions	[161,162]
Hydroalcoholic extracts from Forsythiae fructus rich in Forsythoside A; Phillyrin; Calceolarioside; Rengynic acid	Influenza, RSV	Chito-oligosaccharides	[163]
Extracts from Forsythiae fructus rich in 3,5-dicaffeoylquinic acid, 3,4-dicaffeoylquinic acid, neochlorogenic acid, chlorogenic acid, cryptochlorogenic acid, isoforsythoside, forsythoside A, forsythoside B	Influenza, RSV, HIV, NDV	Chito-oligosaccharides	[164]
Andrographolide	DENV, CHIKV, HBV, HCV, HSV1, EBV, HIV HPV16, pseudovirus, influenza.	Microspheres; nanosuspension and self-nanodispersion; nanoparticles, SMEDDS, inclusion complex	[165,165]
Anthocyanin-rich extracts (from Mulberry) and purified compounds (i.e., delphinidin, cyanidin, and pelargonidin)	Influenza virus, HSV type 1 and 2, RV, Adenovirus 36, BT2, T4 and simian rotavirus SA-11, HAV, FCV-F9 and MNV-1, HCV, WNV, DENV, and ZIKV.	Micro/nanoencapsulation systems, nano/micro-gels, spray-drying and freeze-drying, electrohydrodynamic encapsulation, emulsification and liposomal encapsulation	[166,167,168] [169]
Curcumin	ZIKV, CHIKV, norovirus, Influenza, DENV type 2, RSV, HBV, HCV, HPV, HIV, CMV, EV71.	Nanoparticles, solid dispersion, SNEDDS, SMEDDS, lipid carrier, copolymeric and mixed micelles, exosomes	[170,171]
Water extract from Panax ginseng root and punicalagin	HSV types 1 and 2	Hydroxypropyl methylcellulose (HPMC) hydrogel	[172,173]
Water extract from Panax ginseng root and punicalagin	Influenza A virus (strain A/PR/8)	Ultra-sonication-assisted silver nanoparticles	[172,173]
Hydroalcoholic extract from Elderberry and Eucalyptus	Influenza virus	Oil-in-water (o/w) and water-in-oil (w/o) emulsions	[174,175]
Hydroalcoholic extract from Elderberry and Eucalyptus	Common cold	Encapsulation (BerryPharma^®^ brand)	[174,175]
Coumestrol	HSV types 1 and 2	Lipid nanoemulsion based on dioleylphosphocholine	[176]
Quercetin	JEV, influenza A, EBV, MAYV, RV, HCV	Nanocrystal, nanoparticles phytosome, nanoliposome, mixed micelles, SNEDDS, nanocarrier, nanoemulsion, nanosuspension	[177,178]
Naringenin	DENV, HCV	SNEDDS, solid dispersion, nanoparticles, liposome, nanosuspension, cyclodextrin complex	[178,179,180]
Resveratrol	HIV/AIDS	Polymeric nanoparticles, solid-liquid nanoparticles, self-emulsifying methods, nanosponges, liposomes, emulsion-liposome blends, lipid-core nanocapsules, active lipospheres	[179,180]
Silibinin	Hepatitis C virus infection	Encapsulation based on phytoliposomes	[181]

## 4. The Healing Potential of Medicinal Plants

Medicinal plants are traditional resources that humans use worldwide to fulfill their basic needs, including curative treatments that are fundamental part of the numerous traditional medicinal and health systems [182]. Throughout evolution, humans have studied the biological and medicinal activity of plants and identified their non-toxic products and compounds labeled as phytochemicals. An extensive literature is devoted to research on the isolation and structural elucidation of numerous phytochemicals. In the following paragraphs, the plant extracts and the most important phytochemical classes of various plant foods are presented from a chemical and biological point of view, with particular attention to antiviral activity. The long history that testifies the use of medicinal plants and its extracts for the treatment of various pathologies, represents a fundamental starting point for the study of new alternative drugs to synthetic ones. This section reports some historical notes and important published data regarding the use of herbs and plant extracts for the treatment of various viral diseases.

### 4.1. Herbs Remedies for viRAL Diseases: Historical Notes and General Aspects

Although history teaches us that vaccination is the most effective and powerful method to combat effects of epidemics, the availability of antiviral drugs is also very useful, especially for slowing the spread of new pandemic viruses, thus allowing producers to prepare large quantities of pandemic vaccine. In this perspective, the possibility of developing new antiviral drugs represents a need felt by the whole scientific world and the opportunity that is granted to us by use of natural sources is truly valuable. Traditional or conventional methods have supplied the herbal preparations to the pharmaceutical industries, and this will guarantee a growth in this production by ensuring new principles for the fight against virus-derived diseases [7,183,184]. The initial development of the antiviral drugs was done by European researchers right after the Second World War. It was led by Boots Drug Company from England that examined the action of 288 plants and their respective phytoconstituents against influenza A virus, and at the end of the investigation, 12 plants showed suppressing effects [185].

Plant extracts commonly contain many classes of compounds and among these alkaloids, terpenes and polyphenols. These three classes represent the most widespread groups of secondary metabolites with biological activity, including the antiviral one studied by cell cultures and in vivo testing. Compounds belonging to these classes are often found in nature also in conjugated form. Along with this, various screening programs were instigated for the evaluation of antiviral activity of phytochemicals and the same was done through in vivo and in vitro assays. Overall, in recent and older literature, we find many examples of plant extracts and natural compounds, that exhibit antiviral activity. Relevant examples are the flavonoid glycoside rutin and its aglycon form, quercetin [186,187,188,189].

### 4.2. Plant Extracts

**Figure 5:**
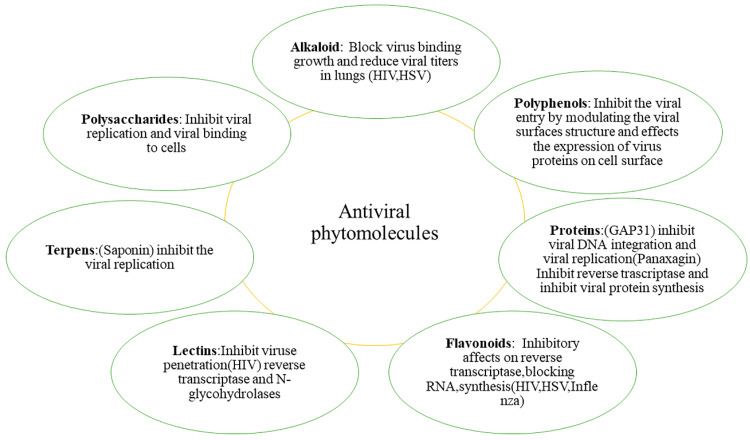
Major groups of phytochemical classes presenting antiviral activities.

To date, there is a lot of evidence confirming that viral infections can be treated by the antiviral drugs obtained by traditional medicine and herbal sources rich in phytochemicals. A collection of the main results relating to antiviral plant extracts was presented in Table 1, while Figure 5 depicts the major phytochemical classes with antiviral activity. Several authors have considered a group of plants or a particular species from which to recover bioactive compounds with antiviral properties. Below are some relevant examples of plants or plant extracts with high antiviral power. Following this strategy, in 1995 McCutcheon and his co-workers screened about one hundred medicinal plants from British Columbia for their antiviral activity. It was found that about 12 herbal extracts had antiviral action, and this included *Rosa nutkana* that was active against enteric coronavirus [190]. The root extracts isolated from *Potentilla arguta* exerted antiviral activity against respiratory syncytial virus. The antiviral activity against parainfluenza virus type 3 was exerted by *Ipomopsis aggregata*. Rota virus related infection can be treated using root extracts of *Lomatium dissectum* [191]. The herpes virus comprises of HSV-1 and HSV-2 both are double stranded linear DNA and comprises of icosahedral proteins as genetic composition. The infection of HSV involves painful reaction and generally can be spread or restricted around the genital area, mouth, skin and eyes. The entrance is based on interaction with glycoproteins that relies on viral envelope and transmembrane receptors of host cells. As such no permanent cure exists, but the viral shedding can be reduced by providing treatment with phytochemicals [18,192,193]. 

The various phytochemicals isolated, identified and tested for the treatment of herpes involves major class of flavonoids and lignans. The type 1 HSV can be treated via extracts obtained from *Verbascum thapsus*, *Cardamine angulata*, *Conocephalum conicum*, *Polypodium glycyrrhiza*, and *Lysichiton americanum*. The traditional medicine tested about 40 different medicinal plants for their antiviral activity against the RNA viruses, DNA viruses, and poliovirus type 1. It was reported that extracts from *Euphorbia australis* and *Scaevola spinescens* had antiviral activity. The liquid extract obtained from *Eleutherococcus senticosus* possessed antiviral activity against human retrovirus and influenza A [16,17,192,194]. In addition, a particular mention deserves the potential antiviral activities reported for essential oils. In this regard, virucidal effects of essential oils extracted from numerous aromatic and herbal plants are well documented on a variety of viruses, such as influenza virus, HSV, HIV, yellow fever virus, and avian influenza. Comprehensive information regarding the antiviral effects of plant-derived essential oils can be found in a recent report by [195].

In an in vitro study on HepG2 cells conducted by Arbab and his co-workers [196], around 60 medicinal plants were taken into consideration against hepatitis B virus. The evaluation of the antiviral power of these plants was carried out by measuring the expression of hepatitis B surface antigen (HBsAg) and hepatitis B virus e antigen (HBeAg). All the 60 plants selected had their potential role in treatment of liver disease and exerted their hepatoprotective and anti-retroviral action. Various extracts like dichloromethane extract of *G. senegalensi* showed their potential role in the treatment of stomach, veneral, respiratory, microbial, dermatological and anti-HSV activity. Another extract which had its potential role in jaundice and hepatobiliary disorders is *F. parviflora*. It exerted its role in anti-HB, jaundice and hepatoprotective action in rats. Extracts from different Acacia species have shown important antiviral activities on HIV and HBV as well as hepatoprotective action. Overall, the antiviral action against HBV is ascribed to numerous phytochemicals such as flavonoids, lignans, terpenoids, alkaloids, anthraquinones and saponins [196].

## 5. Phytochemicals

This section presents the main chemical classes of phytochemicals as antiviral agents and, in some cases, their potential mechanisms of action. In particular, the various phytochemicals and their impact on viral diseases that are still endemic today were reviewed, highlighting in many cases encouraging results in this regard.

### 5.1. Flavonoids

Flavonoids in the form of flower pigments from families of flowering plants/angiosperms contain polyphenols with a skeleton of 15-Carbon atoms [197]. The skeleton consists of C6-C3-C6 system, but in few cases the heterocyclic six-member ring is replaced via 5-membered ring. Since flavonoids comprises of plethora of variously correlated compounds from a chemical point of view, they have been classified into various subclasses based on some chemical characteristics inherent in their structure. Specifically, for the classification of these compounds, the carbon of the C ring on which the B ring is attached was taken into account, as well as degree of unsaturation / oxidation of the C ring [197]. When ring B is connected in position 3 of ring C these compounds are called isoflavones. If the B ring is linked in position 4, they are classified as neoflavonoids, finally if the B ring is linked in position 2, they can be classified into various subclasses which include flavones, flavonols, flavanones, flavanonols, also flavan- 3- oils, anthocyanins, chalcones and dihydrochalcones. Flavonoids are major source of antiviral agents by inhibiting numerous enzymes, this includes xanthine oxidase, Ca2+-ATPase, cyclooxygenase, lipoxygenase and aldose reductase [198].

Flavonoids are one of the most important and studied phenolic compounds. These phytochemicals are plant secondary metabolites and are widely found in different food matrices, such as fruits, vegetables and certain beverages [199]. In the last years, flavonoids have been described as related to several favorable biochemical and antioxidant effects, such as, among the others, those associated with cancer, Alzheimer’s disease, diabetes, and atherosclerosis [200,201]. Besides, flavonoids are important components in several nutraceutical, pharmaceutical, medicinal, and cosmetic applications. In fact, the health-promoting properties of these compounds are usually coupled with their potential inhibition/modulation of key cellular enzymes [202]. They also exert regulatory action on hormones like thyroid, androgen and estrogens [203]. Overall, flavonoids have been studied for their potential antiviral activities and promising results have emerged from both in vitro and even in vivo studies. The next sub-paragraphs summarize the up-to-date evidences for antiviral activity of different flavonoids, together with the cellular and molecular mechanisms of action of these bioactive on viruses.

#### 5.1.1. Anthocyanins

Anthocyanins represent one of the most important class among flavonoids, mainly related to a great range of colors in the plant kingdom [204]. Anthocyanidins, i.e., the aglycones, are characterized by a benzyl ring (A) linked to a heterocyclic ring with oxygen function (C), which is also attached to a third benzyl ring (B). The part C can be linked to parts A and B with three carbon bridges [205]. Besides, the sugar moiety can be found at 3 or 5 positions or both, including monosaccharides, as well as di-saccharides, and tri- saccharides, which could possess acylated side chains. The database Phenol-Explorer reports 71 compounds in different foods, with the highest levels of information available for cyanidin 3-*O*-glucoside and malvidin 3-*O-*glucoside. The antiviral activities of anthocyanins-enriched plants is well documented and reviewed; in this regard, several compounds (mainly glycosidic forms of cyanidin and delphinidin) and have been related to antiviral action against InfV A and B, HSV-1, RV, CV-B1, Adenovirus 36, BT2, T4 and simian rotavirus SA-11, avian InfV, HAV, FCV-F9 and MNV-1 [206]. The previous activities have been described considering different food sources such as, among the others, pomegranate, cocoa, goji berries, grape, berries, and *Solanum* spp. [167]. Although, therefore, many scientific evidence asses the antiviral properties of this class of polyphenolic substances, it should be pointed out, however, that anthocyanins are extremely unstable compounds, highly prone to oxidation mechanisms. Furthermore, their stability is tremendously affected by external factors, such as temperature or pH, which could affect their antiviral activities [207]. In this regard, several technologies (such as those based on nanomedicine) are trying to overcome these limitations to improve the delivery of anthocyanins to the targeted site of action. Intriguingly, some authors demonstrated that encapsulating these phenolics into nanoparticles plays a significant role in improving their antiviral properties [208,209].

#### 5.1.2. Chalcone and Dihydrochalcones

Another important sub-class among flavonoids is represented by chalcones [210]. with antiviral activity. Chalcones are aromatic ketones with two aromatic rings bonded through a three-carbon α, β-unsaturated carbonyl system. Their formation derives from substitution reactions of 1,3-diphenylpropenone and from the compounds derived from it. They are precursors of flavonoids and isoflavonoids, abundantly found inside edible plants. These compounds have been studied as related to the inhibition of human rhinoviruses and plant viruses and the activity relies on substitution patterns. The antiviral activity against tomato ringspot nepovirus (ToRSV) was investigated via Onyilagha and his co-workers using chalcones that are hydroxy and methoxy substituted. The hydroxylation at 2′, 3′, 4′ positions of ring B and at C-4 of ring A promotes antiviral activity against ToRS virus [211].

Deng and coworkers studied the antiviral effects of chalcone 3 [212]. Pharmacophore models were developed for the identification of antiviral properties and for the validation of forty-four compounds that exhibited antiviral activity at different potency rate Dihydrochalcone derived via *Millettia leucantha* possessed antiviral action against the herpes simplex virus (HSV) [213]. In recent literature, antiviral activity has been reported relative to new synthetic chalcone derivatives containing a purine and benzenesulfonamide fraction [213]. The results of this in vivo study confirmed significant anti-TMV and anti-CMV activity related to these compounds. These are the derived products obtained from chalcones by the reduction of the double bonds that disrupted its ability to from normal chalcone pharmacophore. Mars studied a set of synthetically generated chalcones, reported a potential antiviral activity against hepatitis C virus [214]. Finally, dihydrochalcone derivatives from *Millettia leucantha* (Fabacee) was reported to possess antiviral action against the herpes simplex virus (HSV) [215].

Since 1980, another life-threatening issue caused by virus is AIDS due to HIV. Xanthohumol commonly found in hops, serves as a potent inhibitor of enzyme reverse transcriptase thereby inhibiting the process of viral replication [216] The replication of HIV-1 is also inhibited in the peripheral blood mononuclear cells. Buckwold and his coworkers examined antiviral action of a xanthohumol enriched extracts of hops against herpes simplex virus type 2 (HSV-2), bovine viral diarrhea virus (BVDV) and rhinovirus (rhino) [217]. It was found that chalcone 74 from the genus Desmos exhibited potent antiviral activity against HIV. The anti-HIV action has also been described for lycocalcones A 1A, B-76, specifically it has been reported that these compounds, together with 3,3′, 4,4′-tetrahydroxy-2-methoxicalcone, acted against virus by suppressing the TPA-induced HIV promoter by promoting binding to specific proteins [218].

#### 5.1.3. Flavones, Flavanones, Flavonols, Isoflavonoids and Derivatives

Flavones consists of 2-phenylchromen-4-one backbone moiety (2-phenyl 1-benzopyran-4-one), belong to the class of flavonoids and can be obtained from the plant families of *Apiaceae*, *Lamiaceae,* and *Asteraceae*. The phenolic compounds identified and isolated from artocarpus heartwood was responsible for antiviral activity against HSV [219]. Naringin, i.e., chemically 3′,4′-diacetoxy-5,6,7-trimethoxyflavone, showed antiviral activity against picornavirus and respiratory viruses. The structural configuration of methoxy flavones was related to the anti-picornaviral activity [220].

The reduction of double bonds at C4 carbonyl carbon is responsible for the formation of flavanones. Abyssinone II is a naturally occurring flavanone with antiviral activity against HSV-1 that was tested on Hela 5 cells by recombinant α-galactosidase strains of HSV-1 [221]. Dihydroflavonols are flavonoid derivatives that have characteristic hydroxyl moiety at C3 position of a flavanone molecule. They show hepatoprotective and antiviral activity against mycotic infection, auto-immune disease and hepatitis B [222]. Flavonols have a 3-hydroxy-2-phenylchromen-4-one backbone [202]. The database Phenol-Explorer reports 78 compounds included in this sub-class, with the 32% belonging to quercetin and its glycosidic forms, rutin. In this regard, among flavonols, the antiviral effects of quercetin were the most widely investigated [223] together to flavonoid glycoside rutin, to which was ascribed antiviral activity against HSV-1, para influenza 3 virus, HSV-2 and avian influenza virus. Interestingly, quercetin, inhibits the process of replication of various viruses and helps with treatment of pathogenic viral infections like rhinovirus, mayaro virus, influenza virus, adenovirus, respiratory encephalitis poliovirus type 1, HSV-1, HSV-2 and RSV in a dose-dependent manner [186]. Several studies have focused on determining the mechanism of action of quercetin. Hung and coauthors (2015) proposed some mechanisms for the antiviral activity of quercetin against HSV [224], and in particular they considered both the ability of this compound to block virus binding and the ability to penetrate the host cell. Furthermore, in this same research, it was reported that one of the mechanisms by which quercetin could express its antiviral potential towards HSV was its ability to suppress NF-κB activation. Another proposed mechanism of action is by inhibiting the activity of numerous heat shock proteins (HSP). They are produced in response to cellular exposure to stress and are responsible for process of translation via nonstructural protein 5A-mediated viral internal ribosome entry site or Ns5A-mediated viral IRES translation. Another potential mechanism targets inhibition of an enzyme HCV NS3 protease which is responsible for viral replication inside the host cells [186,187,188,189].

Kaempferol is another flavonol fully characterized in both food and plant matrices, presenting interesting biological properties [224]. Kaempferol and its derivatives characterized by acyl substituents showed inhibitory activity against HCMV. Furthermore, the kaempferols of *Ficus benjamina* leaves have shown greater antiviral activity towards HSV-1 and HSV-2 in glycosylated form than as aglycones [225]. Furthermore, the kaempferol derivatives, characterized by a rhamnose residue, showed strong inhibitory activity at the level of channel 3a of the coronavirus, a channel that is important in the complex release mechanism of the virus [225]. Besides, it was reported that kaempferol was one of the flavonoids exhibiting the most potent inhibitory activity against murine norovirus and feline calicivirus [226]. 

Another naturally occurring bioflavonoid is myricetin that exerts action against hepatitis B, coronavirus and influenza virus [227]. In particular, [228] it seems that myricetin is able to influence the ATPase activity of the viral helicase, thus promoting an antiviral action against the SARS coronavirus. Moreover, myricetin was related to an effective inhibitory activity against hepatitis B and influenza virus [223]. Besides, [227] showed that glycosylation might enhance the anti-HIV-1 activity of myricetin, enabling a better internalization of this compound into the cell, then acting on the HIV-1 reverse transcriptase. Flavonoids that act as reverse transcriptase inhibitors, as myricetin (3,3′,4′,5,5′,7-hexahydroxyflavone), baicalein (5,6,7 trihydroxyflavone) and quercetagetin (3,3′,4′,5,6,7hexahydroxyflavone) are abundantly available from natural sources found in nuts, berries, vegetables and fruits. Since various steps involved in the development of rhinoviruses includes viral genome transcriptions and protein synthesis, they can be used in treatment of HIV and Rauscher murine leukemia viruses. Glucuronide of baicalein is baicalin which shows its antiviral activity against dengue virus, enterovirus.

Derivatives of flavonoids that mainly occur via migration of phenyl group from C-2 to C-3 include various compounds as isoflavones, isoflavanones, isoflavans, and isoflavones. Isoflavones were mainly found in family of *leguminosae* and their in vitro microbial testing showed considerable antiviral effect [228]. Isoflavanones differ from isoflavones only due to the presence of a chiral center; naturally obtained prenylated isoflavanones from *Bolus Harms* had noticeable activity against HIV [229]. Isoflavans belongs to the subclass of isoflavonoids without carbonyl groups at C4 carbon that also show antiviral activity.

#### 5.1.4. Flavans and Neoflavonoids

Flavans consist in a 2-phenyl-3,4-dihydro-2H-chromene skeleton. These compounds do not have carbonyl group at 2nd position, such as catechin and epicatechin. The flavans can be divided, in turn, into flavan-3-ols, flavan-4-ols and flavan-3,4-diols. Remarkable biological and pharmacological properties are ascribed to these compounds, and mainly to catechin and its derivatives such as epicatechin, epicatechin gallate, epigallocatechin (EGC) and epigallocatechin gallate (EGCG) are attributed antiviral properties. These compounds are mainly abundant in tea [161,223]. In this regard, tea catechins have been subjected to several studies, mainly as related to the inhibition of influenza virus. Interestingly, a structure-activity analysis of tea catechins demonstrated the pivotal role of the 3-gallolyl group as related to their antiviral activity [223]. Tea catechins have been also reported as potential inhibitors of HIV and herpesviruses [230]. A relevant example of flavans with antiviral activity is provided by the flavan derivatives 7-*O*-galloyltricetifavan and 7,4-di-*O*-galloyltricetifavan present in the methanolic extracts of the leaves of *Pithecellobium clypearia*. [231]. Besides, neoflavanoids are defined as the flavonoid derivatives that comprises of aryl groups attached at C4 position. It includes inophyllum B and inophyllum P with antiviral activity against HIV [232].

### 5.2. Coumarins and Arylcoumarins

Another interesting group of phenolic compounds that contains carbonyl functional group at C2 is coumarin, previously reported to exert antiviral activity [233]. Coumarins are considered good candidates for designing novel antiviral agents. In particular, these compounds have gained great attention in the last years for their correlation with orally bioavailable non-peptidic antiviral agents [233]. The most important coumarin derivatives reported as antiviral drugs belong to the class of 4-hydroxycoumarins, arylcoumarins, pyranocoumarins, furanocoumarins, 3-phenylcoumarins, 4-phenylcoumarins, coumarin-benzimidazoles conjugates, anilinocoumarins, 7-hydroxycoumarin analogues, coumestans, and toddacoumaquinone. Overall, most of the coumarin-derivatives have been linked to the antiviral-activity spectra on HIV type 1, acting as alternative to peptidomimetics. Among these compounds, the most described and presenting a clinical valence are warfarine analogues, tetramers of 4-hydroxycoumarins, khellactone, and calanolide. Besides, there are several studies showing the inhibitory role of coumarin derivatives against infection of Influenza, Enterovirus 71 (EV71) and coxsackievirus A16 (CVA16). Overall, coumarins inhibit proteins essential for viral entry, replication, and infection, acting also as regulators of cellular pathways [234]. One of the most important arylcoumarin is calanolide A that has antiviral activity against HIV-1 and acts via inhibition of non-nucleoside reverse transcriptase inhibitor [231].

### 5.3. Resveratrol (Stilbenes)

Resveratrol belongs to the phenolic class of stilbenes. This phenolic compound characterizes mostly fermented grapes, mulberry, red wine, and peanuts, and it exists as both trans- and/or cis- isomer [235]. In the last years, several health-promoting activities have been accurately documented for this compounds [236], mainly related to its radical scavenging potential. Besides, it acts as prophylactic compound against cancers and viral infections. Resveratrol is certainly the most studied stilbene for its high biological and pharmacological properties and not least its antiviral activity. Several researches have been devoted to the discovery of the mechanisms of action by which this compound exerts this activity on different types of viruses such as influenza, hepatitis C, RSV, HSV, HIV [237]. Significant encouraging results were also obtained towards SARS-CoV-2 [237,238]. Overall, a treatment with resveratrol demonstrated remarkable recession of the viral infection, excepting for multiple sclerosis and hepatitis C infections. Resveratrol was studied in a research conducted by Lin and coauthors in which they confirmed the antiviral activity of the molecule by testing it in vitro towards MERS-CoV, using Vero E6 cells as a cell model. In particular, the authors reported, as a relevant result, a prolongation of the survival of the cell line used after it was infected by MERS-CoV. In particular, this compound caused a reduced expression of nucleocapsid protein (essential for MERS-CoV replication). Besides, resveratrol was found to down-regulate the apoptosis induced by MERS-CoV in vitro, thus demonstrating a very promising role as antiviral agent against MERS-CoV infection. In a recent research the potential anti-rotavirus activity of resveratrol was investigated [239]. In this in vivo study performed on mice, resveratrol was able to reduce the mRNA expression levels of mainly proinflammatory cytokines including interleukin-2, interleukin-10, tumor necrosis factor-α, interferon-γ at the level of the intestinal tissue of animals. These results confirmed the antiviral activity of resveratrol and could be a promising treatment for rotavirus infection.

### 5.4. Other Phenolic Compounds

Regarding other polyphenol classes, the most important compounds presenting antiviral activities can be listed under the class of lignans, tannins and hydroxyphenyl propenes.

#### 5.4.1. Lignans

Lignans are produced by various plant species. From a structural point of view, these molecules can be quite complex as they can be formed by a central nucleus consisting of two or more phenylpropanoid units. Lignans are phytoestrogens with antioxidant activity chemically similar to estrogen [240,241]. Nordihydroguaiaretic acid is lignan obtained from the perspired leaves of *Larrea divaricata* with antiviral activity against numerous viruses including HSV-1, HSV-2, HIV along with human papilloma [216].

Lignans showed diverse pharmacological properties, including their antiviral activities. In a recent review by Cui et al. [242], lignans have been classified in two main groups according to their chemical similarity, being “classical lignans” and “neolignans”. The most studied classical lignans belong to the sub-class of dibenzylbutanes, namely nordihydroguaiaretic acid and niranthin. Nordihydroguaiaretic acid was firstly isolated from the leaves of *Larrea tridentata*. This compound was reported as antiviral agent against numerous viruses including HSV-1, HSV-2, HIV along with human papilloma. Besides, niranthin (firstly isolated from *Phyllanthus niruri* Linn) was studied as related to a potential anti-hepatitis B virus (HBV) activity. Among the lignans, worthy of note is niranthin, capable of inhibiting the replication of HBV DNA and the expression of the antigen of the same virus. Other sub-classes of lignan compounds reported to present anti-viral activities are dibenzylbutyrolactones (such as ATG, yatein, and hinokinin), arylnaphthalenes, aryltetralins, substituted tetrahydrofurans (including lariciresinol), 2,6-diarylfurofurans (including sesamin), and dibenzocyclooctenes (including compounds from the plant Schisandraceae family). Regarding neolignans, the most studied group consists in 1,4-benzodioxane lignans; in this regard, the major compound, namely silymarin, has been documented as active antiviral agent against HCV and other viruses. Overall, extensive and detailed information regarding their mechanism of action and potential target can be found in [242]

#### 5.4.2. Tannins

Tannins are high molecular weight phenolic compounds that have hydroxyl groups, as well as various others, including carboxylic group supports formation of a strong natural complexes. The classification of tannins is based on their ability to undergo hydrolysis. The non-hydrolysable ones are attributable to the structure of flavan-3-oils, and are made up of proanthocyanidins, mainly catechin and epicatechin. This type of tannins can have a different degree of polymerization as well as a different bond arrangement and hydroxylation pattern. Instead, among the tannins that undergo hydrolysis, called precisely hydrolysable, there are gallotannins and ellagitannins, composed mainly of a gallic acid or an ellagic acid, respectively, which by means of ester bonds reach a degree of polymerization even high. Both the very different chemical dimensions, due to the varying degree of polymerization, and their stereochemistry are the main chemical characteristics that define the great chemical diversity within this class of molecules to which their various health properties are related [243].

Indeed, tannins showed a variety of biological effects, including antiviral activity [244]. In this regard, it must be emphasized that these molecules are capable of acting against many viruses including influenza virus A, HIV, HSV and rotavirus. Overall, the most of antiviral activities are reported for ellagitannins (belonging to hydrolysable tannins. The ellagitannins extracted and isolated from *Phyllanthus urinaria* and *Phyllanthus myrtifolius* have been reported to show antiviral activity against Epstein-Barr virus. There is various scientific evidence regarding the antiviral activity of some hydrolysable tannins such as chebulagic acid and punicalagin, in fact the inhibitory action on HCV is ascribed to them. Furthermore, through in vitro experiments, the inhibiting action of strictinin on the replication of the human, swine and duck influenza A virus has been highlighted. [245]. Ellagitannins isolated from *Tuberaria lignosa* inhibited HIV’s entry into MT-2 cells, likely suppressing HIV replication by acting on reverse transcriptase [246]. Moreover, other authors have reported that ellagitannins act by inhibiting the HIV-1 protease and HIV-1 integrase enzymes [244]. Overall, ellagitannins from plants belonging to *Phyllanthus* present antiviral activity against Epstein-Barr virus [244].

#### 5.4.3. Hydroxyphenylpropenes

Regarding other classes of phenolics, 6-gingerol (belonging to the phenolic sub-class of hydroxyphenylpropenes) is a phytochemical found in fresh ginger rhizome (*Zingiber officinale*). 6-Gingerol has been previously described as a promising drug candidate to treat various diseases associated with inflammation, cancer, and viral disease [247]. 6-gingerol possesses antiviral activity against human RSV. Recently, Rathinavel et al. (2020), with the aim to develop structure-based drugs from photochemical compounds and to search for inhibitors against the viral proteins of the corona virus (SARS CoV-2), revealed that 6-gingerol could act as a promising drug of choice to treat COVID-19 disease [247]. In fact, the molecular docking studies showed that 6-gingerol possessed excellent pharmacokinetic properties with the highest binding affinity ranging from −2.8764 KJ/mol to −15.7591 KJ/mol with various COVID-19 viral protein targets.

#### 5.4.4. Curcumin

Yang, M. et al. [248] reported an antiviral activity of curcumin against norovirus in a specific cell culture infection model. In particular, this compound was able to reduce the infectivity of norovirus by 91%. In a recent study, [249] showed that curcumin promoted the blockade of APE1-mediated redox function, and that through this mechanism it was able to inhibit the replication of herpesvirus (KSHV) associated with Kaposi’s sarcoma. Prasad and coworkers (2015) in a review article reported many scientific evidences that Curcumin was effective in multiple steps of HIV infection and multiplication [250].

### 5.5. Terpenoids and Carotenoids

#### 5.5.1. Terpenoids

Terpenoids are diverse class of naturally occurring organic chemicals with various biological properties [251,252,253] that are also known as isoprenoids. Terpenoids can be considered derivatives of mevalonic acid consisting of isoprene units. These molecules are widespread in nature and are usually extracted from plants. The gibberellins, components of this class, are essential in the growth and development of plants, while the carotenoid compounds are mainly related to photosynthetic processes. The phytoalexins, on the other hand, are important as they regulate the defense mechanisms of plants towards the environment. Several volatile terpenoids are variously used, for example menthol and peryl alcohol are used to produce spices, aromatic substances and cosmetics, while pyrethrins and limonoids are used as pesticides or industrial raw materials. Some compounds with a sesquiterpene character such as farnesene or bisabolene and monoterpenic such as pinene and limonene, can be considered fuel precursors. Generally, terpenoid substances are classified according to their chemical structure, and specifically, according to the number of isoprene units. So, we have monoterpenes (C10), sesquiterpenes (C15), diterpenes (C20), triterpenes (C30), tetraterpenes (C40) and polyterpenes (C > 40). It should also be taken into account that these compounds can also occur as oxygen-containing derivatives such as alcohols, aldehydes, carboxylic acids, ketones, esters and glycosides.

A specific group of terpenoids, namely the pentacyclic triterpenoids, composed by three terpene units was found to exert antitumor, anti-inflammatory, and antiviral activities [254]. In this regard, oleanolic, betulinic, and ursolic acids are representative pentacyclic triterpenoids showing a broad antiviral spectrum. Moreover, glycyrrhizin (and its analogues) has been studied as anti-HIV agents according to their different target (i.e., entry inhibitors, reverse transcriptase inhibitors, protease inhibitors, maturation inhibitors, and bifunctional inhibitors). The antiviral activity of compounds belonging to the monoterpenes class has been extensively evaluated and some components such as isoborneol and borneol, have shown a high activity towards HSV-1. In this regard, isoborneol was found to have a total inhibition of HSV-1 replication already at a concentration of 0.06% [255]. Besides, terpenoids have been described also as anti-influenza, anti-SARS, anti-human enterovirus 71, anti-EBV, anti-HBS agents. Comprehensive information regarding their modes of action can be found elsewhere [254].

#### 5.5.2. Carotenoids

Carotenoids are classified under the category of tetraterpenoids, they are composed of hydrocarbons due to the association of several isoprene units. The carotenoids are derived from polyene chain with four carbons that serve as its backbone. These compounds are characterized by a system of conjugated double bonds, which is mainly responsible for some of the reported bioactive properties [256]. They can be classified as xanthophylls consisting of oxygen, e.g., lutein, zeaxanthin. On the other hand, carotenes can be defined as the unoxygenated form of carotenoids [257]. These molecules have been associated with several health benefits [256,258]. However, little information exists regarding the potential anti-viral activity of carotenoids. In previous works, lutein showed antiviral activity against hepatitis B, since it inhibited the transcription of the virus [259]. In recent research, it was found that a C50-series carotenoid, bacterioruberin, was more effective than conventional drugs commonly used to fight HCV and HBV in mononuclear blood cells of infected patients. Specifically, this molecule appears to inhibit the antiviral activity of HCV RNA and HBV DNA polymerases, thereby ensuring the suppression of HCV and HBV replication.

### 5.6. Alkaloids

Alkaloids have heterocyclic ring containing nitrogen constituent and are synthesized in the plants during amino acid pathway. They can be classified based on their chemical structure. For example, those that contain an indole structure are known as indole alkaloids. Hence, alkaloids can be grouped into different classes, alkaloids with tropane or indole nucleus can be had as well as terpenoid and steroid structures. Additionally included in this classification are the quinolinic and isoquinolinic structures, and again the pyridines, pyrrolidines and finally the pyrrolizidines. [260]. These compounds are generally produced by many plant species, mainly flowering plants. Many testimonies of their use as a natural remedy have been reported for millennia in folk medicine. Since the twentieth century, however, single alkaloids with pharmacological properties extracted and purified mainly from plant sources have been produced on an industrial scale. Many alkaloid substances are part of the human diet, being present in both food and drinks. The plants in the human diet in which alkaloids are present are not only coffee, cocoa or tea but also some Solanaceae such as tomatoes and potatoes [261]. About 36 alkaloids isolated from *Catharanthus lanceus* or *Catharanthus roseus* were evaluated for antiviral activity against polio type III virus, and the most effective amongst all was pericalline. The piperidine ring and free hydroxyl groups present in the heterocyclic structure of this alkaloid were responsible for antiviral activity against HIV infection [262,263]. Berberine is a natural alkaloid with an isoquinoline structure present in various medicinal plants, such as *Berberis vulgaris*. A documented antiviral activity has been ascribed to this alkaloid. Of interest, this alkaloid is active against herpesvirus, influenza virus and respiratory syncytial virus as widely reported in the literature. Its antiviral activity, described against SARS-CoV and other coronaviruses, could represent a good way to verify its effectiveness against the new pandemic SARS-CoV-2 coronavirus. Specifically, it has been reported that this compound may represent a remedy for severe acute respiratory syndrome coronavirus (SARS-CoV) infection, the causative agent of SARS respiratory disease belonging to the *Coronaviridae* family. As we know, the SARS-CoV proteins nsp1, nsp2, nsp7, spike and nucleocapsid are responsible for the activation of NF-κB, a protein involved in the control of cellular processes such as inflammatory and immune responses. When the SARS-CoV virus causes disease (SARS-CoV), it happens that this infecting agent is able to regulate the expression of pro-inflammatory mediators such as TNF, CCL2 and CXCL2, at this level berberine is able to inhibit the NF-κB signaling pathway for which it is plausible to imagine its antiviral action against coronavirus infection [264,265]. Yan and co-workers (2018) reported an antiviral effect of berberine against influenza H1N1 virus [266]. The same authors demonstrated that berberine promotes the inhibition of influenza virus replication in A549 human lung adenocarcinoma cells and in mouse lungs by suppressing infection, in particular by inhibiting the expression of TLR7 and NF-κB. Some scientific evidence has reported that low micromolar concentrations of berberine are capable of inhibiting the replication of different HCMV strains. In particular, in the work carried out by Luganini and co-workers [98] it was shown that this alkaloid interferes with the transactivating function of the HCMV Immediate-Early 2 (IE2) protein. This protein is the most important regulator of HCMV and represents a transcriptional activator of viral and cellular gene expression. Other alkaloids widely studied for their antiviral activities are leurocristine, periformyline, perivine, vincaleucoblastine, michellamines D/F, homonojirimycin, deoxymanojirimycin, castanospermine, australine, sesquiterpene, 5-hydroxynoracronycine, acrimarine F, columbamine, and palmitine. Detailed information regarding their targets are fully explained by [267]. Tomatidine is an aglycone metabolite of tomatine, a natural steroidal alkaloid derived mainly from green tomatoes, with marked biological activities such as anti-cancer, anti-inflammatory and capable of stimulating muscle hypertrophy against atrophy and weakness in aged skeletal muscles [268,269,270]. Diosa-Toro et al. [271] studied the antiviral properties of tomatidine against DENV, Zika virus (ZIKV) and WNV [271]. In this study, these authors highlighted potent anti-DENV activity in Huh7 cells of human hepatocarcinoma. Furthermore, the antiviral activity of this alkaloid towards DENV was confirmed in A549 cells of the alveolar epithelium of adenocarcinoma while less potent antiviral activity towards ZIKV was observed and no antiviral effect was towards WNV. Of interest, it was the confirmation of anti-DENV activity even when tomatidine was added 12 h after infection. Recently, [272] reported that tomatidine, can be considered a potential anti-viral against chikungunya virus (CHIKV). Besides, other two compounds, namely solasodine and sarsasapogenin (i.e., two structural derivatives of tomatidine) showed also antiviral activity towards CHIKV, although lower when compared with tomatidine.

### 5.7. Organosulfur Compounds

Another large family of active compounds present in the plant world is represented by glucosinolates. They are present in the genus Brassica (Cruciferous), and represent the first precursors of isothiocyanates. In fact, glucosinolates undergoing chemical or enzymatic hydrolysis are able to generate isothiocyanates and thiocyanates in turn molecules with important biological activities. Overall, the organosulfur compounds group includes not only isothiocyanates, but also indoles, allylic sulfur compounds, and sulforaphane. These compounds are widely known for their unique health-promoting properties. In this regard, their antioxidant antimicrobial and anti-inflammatory activities are already known. These compounds are particularly active against chronic diseases, especially in light of the beneficial effects due to their ability to reduce the level of low-density lipoproteins and carcinogens or toxic agents. Many vegetables belonging to the Allium and Brassica genus such as garlic, onion and broccoli, are primary sources of organosulfur compounds. Plants from *Allium* family synthesize some major classes of antiviral compounds. The main limitation for the use of organosulfur compounds is their pungent odor together with the chemical instabilities, which are characteristics mainly attributable to diallyl sulfides. However, some compounds with a less pronounced odor compounds, such as S-allyl cysteine or S-allyl-mercapto-cysteine are also important constituents of garlic and showed strong bioactive properties [92]. Organosulfur compounds like allicin, diallyl trisulfide and ajoene are the major phytochemicals which impart antiviral property to garlic. In particular, ajoene, allyl alcohol, and diallyl disulfide are reported to act against HIV infected cells [273]. It should also be taken into account that allicin is able to cross the phospholipid bilayer of the cell and this strengthens its ability to inhibit the spread of the virus in the host [92].

### 5.8. Poly-Acetylene and Polysaccharides

#### 5.8.1. Poly-Acetylene

Polyynes are organic compounds with alternative single and triple bonds. Poly-acetylene constitutes under hydrocarbon category have the characteristic feature of absorbing the UV light of wavelengths [274]. Phenylheptatriyne or PHT is poly-acetylenes obtained from leaves of *Bides pilona* and it exerts antiviral effect that alters with exposure towards UV light [275]. Poly-acetylenes are reported to inhibit CMV herpes virus without causing any substantial changes to the DNA and by cell-mediated surface activity [275].

#### 5.8.2. Polysaccharides

Besides, polysaccharides are complex carbohydrates composed of galactose, glucose and xylose with general formula C_n_(H_2_O)_n_, where value n lies between 200–2500 units. The sulfated polysaccharides that showed antiviral activity against numerous animal viruses can be isolated from red algae with naturally sulphated cellular wall [276,277]. Overall, among the most important polysaccharides as related to anti-viral activities we found galactans, alginates, and fucans [277,278,279,280,281]. Overall, galactans, alginates and fucans must be considered among the most important polysaccharides with antiviral activity [282,283,284,285,286]. In an in vivo study [287] it was reported that carrageenans (molecular weight: 3, 5 and 10 kDa), and its acetylated and sulphate derivatives, were capable of acting against the influenza virus and HIV by means of depolymerization and sulfation. This meant that small molecules such as sulfated polysaccharides could be considered as potential antivirals [278].

Huleihel and coworkers evaluated the effects of polysaccharides obtained from numerous species of red algae. *Porphyridium species* exerted potential antiviral action against HSV-1 and HSV-2 along with Varicella zoster virus [240,278,279]

### 5.9. Antraquinones and Gingerol

Anthraquinones (9,10-dioxoanthracenes) are an important class of natural/synthetic compounds with a great range of applications [279]. The most studied compounds as related to anti-viral activity are chrysophanol, emodin, and aloe-emodin [280]. Anthraquinone based compounds includes chrysophanic acid, or chrysophanol, which is chemically 1,8-dihydroxy-3-methylanthraquinone and is extracted and isolated from Australian-based medicinal plant *Dianella longifolia*. Chrysophanol showed antiviral activity against poliovirus through in vitro assays, mainly inhibiting the replication of poliovirus types 2 and 3 in vitro. Compared to other anthraquinone compounds, chrysophanol showed the highest antiviral activity against type 3 poliovirus [281]. Emodin is anthraquinone used for the treatment of SARS-associated coronavirus which is obtained from genus polygonum and acts by inhibiting and blocking interactions of the coronavirus with ACE receptors [282]. Recently, anthraquinones have been proposed as agents capable of counteracting SARS CoV-2 as, as reported in a molecular docking study, they are potentially capable of inhibiting the main protease of this virus. However, this property would be present in several anthraquinones since they are able to establish non-covalent bonds in the area adjacent to the active site with catalytic dyad HIS41 and CYS145. However, the inhibitory potential of these compounds was lower when compared to the FDA approved drug remdesivir. The data obtained from this research on the antiviral role of anthraquinones, highlighted that the alterporriol Q could be considered among the anthraquinone compounds the most promising inhibitor of SARS-CoV-2 Mpro. Gingerol is product derived from ginger and mainly employed for the treatment of sore throats and common cold. It is used as a major constituent in ayurvedic formulations [283].

### 5.10. Salicylic Acid and Chlorophyllin

Salicylic acid is regarded as naturally colorless and crystalline organic acid, serving a major role as plant hormone [284]. Studies have reported that it can have potentiating action against wide range of pathogens and its subtypes. Salicylic acid can act via inhibiting the stages of replication, long distance movement and cell-to-cell movement in viruses. It was reported by the evidence that it stimulates the downstream pathway that can lead to induction of mechanism of resistance based on RNA interferences [285,286]. It has been attributed to a synthetic derivative of chlorophyll, chlorophyllin (CHLN), an antimutagenic activity against numerous environmental contaminants. Botelho and co-workers (2004) reported the ability of this compound to prevent nuclear fragmentation (NF) in polio virus-infected HEp-2 liver cells. [287].

## 6. Plant Foods in the Preparation of Antiviral Drugs

Many plant foods have been deeply investigated for their immunomodulatory, antiviral properties as well as other relevant biological activities. In recent months, following the COVID-19 pandemic, many studies have been conducted on humans using natural extracts from plants and plant foods. Below, we report some commonly used and less known food plant species, to which a series of scientific evidence are ascribed confirming the antiviral activity strictly correlated to the presence of the phytochemical’s classes described above.

### 6.1. Ginseng

Ginseng obtained from Panax ginseng has immuno-modulatory effect with antiviral property against RSV that was investigated by Seok and his coworkers [288]. Ginseng inhibits viral replication and thus helps in the improvement of epithelial cells of the lungs. Another possible mechanism includes inhibition of inflammatory cytokines that are produced via production of reactive oxygen species. The Korean red ginseng is responsible for the inhibition of herpes simplex type virus. Its direct antiviral effects include inhibition of viral attachment, its penetration and replication to the host cells and enhancements of their immunity [288,289,290].

### 6.2. Basil and Oregano

Basil is a component of *Ocimum basilicum* L. and its antiviral activity was first investigated by Chiang and his co-workers. Basil is known as sweet basil and its purified extract was investigated against viral infections that are related to DNA viruses. The bioactive compounds include linalool, apigenin and ursolic acid that had antiviral activity against enterovirus 71, adenovirus (ADV), hepatitis B virus and HSV. Ursolic acid had highest antiviral activity against adenovirus, enterovirus and herpes simplex viruses [291].

Oregano belongs to the herb of mint family and has antiviral and medicinal activity due to the presence of eatable carvacrol and other bioactive compounds. Gilling et al. (2014) demonstrated the antiviral activity of carvacrol that was active against murine norovirus (NMV), so it can be used in foods as preservative and as antiviral agent for preventing infections. Another study was based on the investigation of antiviral action of Mexican oregano, and it was found that it has antiviral effects against human respiratory syncytial virus (HRSV) [292,293,294].

### 6.3. Fennel

Previous studies have shown the existence of numerous valuable compounds that are obtained from *Foeniculum vulgare* Mill. (fennel), including flavonoids, volatile compounds, fatty acids, phenolic compounds, and amino acids. The collected data demonstrated their efficacy in many pharmacological properties done in vitro and in vivo, with reported anti-inflammatory, antimicrobial, antinociceptive, antiviral, antimutagenic, antipyretic, antispasmodic, cardiovascular, apoptotic, antithrombotic, antitumor, hepatoprotective, chemo modulatory, hypoglycemic, hypolipidemic action. Orhan and colleagues focused on the antiviral function of essential fruit oil from Fennel. Most of the oils exerted significant antiviral activity against HSV-1 when administered in range from 0.8 –0.025 μg/mL, but were less successful against PI-3 in the range from 1.6–0.2 μg/mL [295,296].

### 6.4. Garlic

Garlic (*Allium sativum* L.) has known antiviral activity against human papilloma virus. In placebo-controlled trials it was confirmed that apical application of chloroform garlic extracts resulted in adequate removal of skin warts without recurrence even after 3–4 months. Cytomegalovirus, herpes simplex virus 1, rhinovirus, herpes simplex virus 2, HIV, viral pneumonia, and rotavirus have been tested in a few studies with garlic [92,297]. Organosulfur compounds as allicin, trisulfide diallyl, and ajoene showed activity as antiviral agents. In the case of HIV, ajoene is thought to function by inhibiting processes based on the integrin, while allyl alcohol and diallyl disulfide also have proven effeteness against cells infected with HIV. However, opposite to common belief there are inadequate clinical trials that measure impact of garlic on common cold prevention or treatment [92,297]. Recently, [298], reported that 17 organosulfur compounds identify by GC-MS in garlic essential oil, showed strong interactions with the amino acids of the ACE2 protein (involved in coronavirus resistance) and the main protease PDB6LU7 of SARS-CoV-2. This result is of particular interest, thus suggesting that garlic essential oil could be a valuable natural anti-virus source, able to prevent the invasion of coronavirus into the human body [299]

### 6.5. Ginger

Ahmed et al. (2017) found that ginger may have antiviral activity against avian influenza virus (H9N2), but further confirmations were required. Additionally, aqueous extracts of clove and ginger showed promising results for the prevention of food-borne viral contamination, while other study reported that fresh vs. dried ginger is more potent against HRSV-induced plaque formation on airways epithelium by blocking and internalizing viral attachments [300,301]. Schnitzler et al. (2007) examined in vitro studies of basic ginger (*Zingiber officinale*), hyssop (*Hyssopus officinalis* L.), thyme (*Thymus vulgaris*, L.), and sandalwood (*Santalum album* L.) essential oils and their activity against HSV-1. Essential oils showed high levels of antiviral activity against KOS and HSV-1-resistant acyclovir-sensitive strain, and reduced plaque formation [301].

### 6.6. Peppermint

Peppermint is derived from *Mentha x piperita* and consists of phenolic components including rosmarinic acid and flavonoids such as hesperidin, luteolin and eriocitrin. The volatile components are made of essential oils called menthol and menthone (principal volatile components in the essential oil) that have antimicrobial, antiviral, and antioxidant activity. Li and coworkers (2017) explored the anti-inflammatory, antiviral, and antioxidant activity of *M. piperita* leaves’ ethanolic extracts (MPE) [302]. Here MPE contained high phenolic acid and flavonoid contents and demonstrated free-radical scavenging activities and high antiviral selectivity activity against RSV (breathing syncytial virus). Furthermore, a significant reduction of the nitric oxide (NO), interleukin (IL)-6, tumor necrosis factor alpha (TNFα), and prostaglandin E2 (PGE2) in lipopolysaccharide-stimulated RAW 264.7 cells, were reported [302,303].

### 6.7. Sambucus

*Sambucus nigra* L. or commonly called elderberry has been extensively used herb to treat influenza and colds. The research by Kinoshita and colleagues (2012) assessed antiviral activity of concentrated elderberry juice (CJ-E) on human influenza A virus, and results showed that CJ-E exerted relatively strong effect on mice infected with influenza virus [304]. However, its antiviral activity against influenza virus in vitro was not very remarkable. In order to evaluate the antiviral activity of the juice in vivo, it was subjected to ultrafiltration and anion exchange chromatography. An interesting result of this experiment was the complete suppression of virus replication in bronchoalveolar lavage fluids (BALF) following oral administration of previously separated and high molecular weight CJ-E fractions to influenza virus-infected mice. To complete these results, data relating to the increase in influenza virus-specific neutralizing serum antibodies and the increase in secretory IgA in BALF and feces were also added. One of the separate high molecular weight fractions, fraction II rich in acidic polysaccharides, was capable of exerting a good defense against virus attack. The authors concluded that the treatment with CJ-E could be considered positively in terms of immune response and with respect to the ability to prevent viral diseases. Meta-analysis synthesized the results of elderberry supplementation on causes of the upper respiratory symptoms while controlling for vaccination status and underlying pathology [305]. It was found that supplementation with elderberry has considerable ability to reduce upper respiratory symptoms and can be safer alternative to prescription medications.

### 6.8. Licorice

Licorice (**Glycyrrhiza uralensis**) is a commonly used herb for centuries in traditional Chinese medicine, containing approximately 20 triterpenoids and 300 flavonoids. Numerous studies demonstrated that among others, these metabolites have antimicrobial, antiviral, anti-inflammatory, and antitumor pharmacological functions. Licorice has an antiviral property against hepatitis C virus, herpes simplex virus, Coxsackievirus B3 and A16, influenza virus H5N1, enterovirus 71, rotavirus and human respiratory syncytial virus 30. Glycyrrhizin isolated from *G. glabra* and *G. uralensis* is one of the principles tested on patients with SARS-CoV infection at the time of the outbreak in 2002 [306]. The authors of this study showed that glycyrrhizin effectively inhibited SARS-CoV replication on Vero cells in addition to blocking the adsorption and penetration of the virus. Furthermore, glycyrrhizin possesses a powerful activity against the influenza A virus (H5N1), reducing the ability of H5N1 to influence the production of chemokine and interleukin (IL-6) [307]. Researchers explored the antiviral properties of licorice root extracts and found that extracts had a higher anti-HIV activity than water extracts. On the other hand, the water extract, in particular the flavonoid-rich fraction, showed a higher antiviral activity against HSV than the alkaline extract. The flavonoid fraction showed greater cytotoxicity for human oral squamous cell carcinoma lines compared to normal cells [308]. Another study reported on reduction of HRSV-induced plaque in respiratory mucosal cell lines by licorice root [309]. Extracts were selective against HRSV plaque in respiratory mucosal cell lines and showed antiviral activity against infection on airway epithelial cells.

### 6.9. Rosemary

Rosemary (*Rosmarinus officinalis* L.) is a medicinal plant that is native to Mediterranean but cultivated in all parts of the world. Rosemary has bioactive phytomolecules with anti-inflammatory, antimicrobial, antiproliferative, antioxidant, and antitumor pharmacological activities. Antiviral activity is mostly derived from chemical compounds as rosmarinic acid and eucalyptol, while it was earlier reported that triterpenoids, as oleanolic acid (OA) and its analogues, were carriers of the antiviral, anti-inflammatory, antitumor and anticancer properties. OA is relatively non-toxic and conversion of its functional hydroxyl and carboxylic acid groups results with active and potent antiviral compounds. Synthesized compounds had antiviral activity against HIV via inhibition of HIV-1 replication. They also impeded entry of influenza virus to host cells by the inhibition of binding of hemagglutinin protein. There were also few reports of potential OA reactions against hepatitis B virus [310], and HSV-1 and HSV-2 [311]. Battistini and his colleagues (2019) investigated the antiviral activity of oils in soft fruits against hepatitis A virus, showing that rosemary cineole was the most potent for reducing viral infection [312].

### 6.10. Moringa

Moringa (*Moringa oleifera* Lamarck) belongs to the Moringaceae family and although its origins are the regions of northern India, today it is possible to find it in many geographical areas, especially in the warmer regions of the globe such as tropical and subtropical areas. [313]. Moringa represents an important multi-tasking crop [314] when considering medicinal and nutritional uses. It is characterized by several bioactive phytochemicals, including beta-carotene, proteins, vitamins and variety of phenolics [8,9,315]. Many biological and pharmacological activities have been reported for various parts of this plant, especially leaves, fruit, roots, seeds and pods that have shown interesting cardiac and circulatory activities [316], or antitumoral activity [317], and anti-inflammatory property [318]. In addition, antidiabetic [319] and hepatoprotective [320] activities have been documented.

To date, in the context of African Traditional Medicine, this medicinal plant is among the most used by herbalist and medics to treat or manage disease of people living with HIV/AIDS (PLWHA). There are many positive feedbacks on its effectiveness in improving quality of patients’ lives and the ability to induce improvements of HIV/AIDS disease. From this point of view, to evaluate antiretroviral activity of different extracts of M. oleifera, Nworu and collaborators [321], used a specific antiviral screening technique, demonstrating that three different extracts of M. oleifera were able to inhibit infectious power of HIV-1 lentiviral particles. In a recent research, [322] the antiviral activity of aqueous extracts of M. oleifera leaves was evaluated on Huh7 cell line. In particular, these extracts were able to modulate the pgRNA levels and HBsAg secretion positively in Huh7 cells transfected with genotypes C and H of HBV. In a study published in 2017, [323], the authors assessed the potential of *M. oleifera* and *Rosmarinus officinalis* Lam. against herpes simplex viruses. Interestingly, the results showed that the aqueous extract of *M. oleifera*, at a concentration of 200 μg/mL, inhibited herpes simplex type 1 and type 2 by 43.2% and 21.4%, respectively. Instead, the 100 μg/mL rosemary extract inhibited herpes simplex type 1 by 18.9% and was not active against HSV-2, so the authors concluded that *M. oleifera* extract was more effective than rosemary as antiviral agent against herpes simplex viruses. In an in vitro investigation on BHK-21 cell line, the ethanolic extract of *M. oleifera* leaves, at different concentrations, showed significant anti-Food and Mouth Disease Virus (FMDV) activity. Specifically, the findings indicated that six concentrations 1 μg/mL up to 100 μg/mL were safe for the considered cell culture (cell survival > 50%) and exhibited significant antiviral activity. The authors conclude that probably Niaziminin or another member of the thiocarbamate group, could be responsible for the reported antiviral activity. To date, also considering the data shown above, many authors seem to agree that Moringa can be considered a very promising alternative to conventional antiviral drugs.

### 6.11. Pomegranate

Pomegranate (*Punica granatum* L.) belongs to the family of Punicaceae and is widely cultivated in many tropical and subtropical countries. Overall, many studies reported health-promoting properties (including antioxidant, anticancer, and antimicrobial) of pomegranate peel and fruit extracts [324]. Moreover, the main components responsible for antiviral activity of pomegranate are four major polyphenols, namely ellagic acid, caffeic acid, luteolin, and punicalagin [325]. Interestingly, pomegranate peel (considered as agro-food waste), is also a great source of different flavonoids with both antibacterial and antiviral activities. The antiviral activity of *P. granatum* extracts against HSV-2 has been investigated by Arunkumar and Rajarajan in a recent paper [326]. Authors found a significant inhibitory activity of tfruit peel ethanolic extract, with punicalagin showing 100% anti-HSV2 31.25 μg/mL and strongly interacting with targeted proteins of HSV-2 (as revealed by molecular docking analysis). Moeover, [327] studied the anti-influenza virus activity of total phenolics characterizing pomegranate peel extract and other fractions. The authors showed that the crude peel extract and its *n*-butanol and ethyl acetate fractions were characterized by the higher inhibitory effects against influenza A virus. In particular, the authors reported IC50 values of 6.45, 6.07 and 5.6 μg/mL in MDCK cells, respectively, with a significant reduction in the production of virus in a dose-dependent manner. Punicalagin and pomegranate peel extract in conjunction with zinc (II) salts were tested against HSV in another in vitro study conducted in epithelial Vero host cells [328]. The antiviral action of the extract against HSV-1 in association with different concentrations of ZnSO4 confirmed the ability of this association to fight the virus by enhancing their virucidal activity by over five-fold. From the studies available in the literature, it therefore appears that antiviral activity, especially on HSV-1 and HSV-2, may be closely related mainly to high concentrations of punicalagin.

### 6.12. Blackberry and Other Berries

The blackberry is an edible berry of Rosaceae family, genus Rubus. Although it is a fairly common fruit, its main production is in North America, Europe, Asia, South America, Oceania, Central America and Africa [329]. The fruits of this plant are rich in biologically active substances and class of polyphenols is very well represented with large quantities of phenolic acids such as gallic acid, flavonoids such as quercetin, complex structures such as tannins and ellagitannins as well as anthocyanins and cyanidins. [330]. Besides the phenolic compounds, this fruit is also rich in carbohydrates (such as glucose, fructose, and sucrose), organic acids (such as malic acid), and minerals (mainly potassium). However, it is important to highlight that chemical composition is strictly related to several factors, such as variety, growing conditions, stage of ripeness, harvest and storage conditions. Regarding the potential antiviral activity, [331] evaluated antiviral effects of blackberry extracts against HSV type 1; the authors demonstrated that blackberry extract (≥56 µg/mL) inhibited the early stages of HSV-1 replication in oral epithelial cells by >99%, showing potent virucidal activity. However, there are few other available evidences in scientific literature regarding the exploitation of blackberry extracts as antiviral agents. Therefore, further research in this sense seems to be worthwhile. On the other hand, other available reports on different wild berries showed that strawberry and raspberry (*Rosaceae*) together with bilberry and lingonberry (*Ericaceae*) extracts are potential inhibitors of several viruses that are important human pathogens. In this regard, [332] has been demonstrated that the previously reported plant extracts (according to their specific anthocyanin composition) were all able to inhibit the replications of both CV-B1 and influenza A virus.

## 7. Drug Delivery Strategies of Phytochemicals with Antiviral Activity

As highlighted in the previous sections, phytochemicals extracted from plant foods could represent basis for a natural and alternative pharmacotherapy for treating several viral diseases. Therefore, it is important to consider potential delivery applications of these plant extracts. In order to formulate natural antiviral agents able to overcome the multiple biological barriers existing, thus successfully reaching their intended site(s) of action. In the last years, several promising strategies have been optimized to face the delivery of poorly soluble phytochemicals from plant foods, in order to improve their systemic bioavailability and clinical outcomes [333]. However, the physicochemical properties of the selected natural phytochemicals mainly determine the possible delivery issues. In particular, the most important properties to be considered are (a) log P (lipophilicity), (b) melting point, (c) phytochemical structure and (d) molecular weight. Overall, most widely used delivery systems to date are those that use phytosomal structures, elegant techniques such as self-microemulsifying drug delivery systems (SMEDDS) and self-nanoemulsifying drug delivery systems (SNEDDS), as well as microspheres, nanoparticles, hydrogels, transferosomes and ethosomes). In this sense, microencapsulation of polar or apolar bioactive compounds represents today an effective solution to overcome these problems. This technique is able to preserve and stabilize phytochemicals from physical-chemical stresses, protecting them from adverse environmental conditions and thus increasing the potential bioavailability.

Furthermore, several new protected delivery systems have been developed in the preparation of functional food. These new systems can be of mechanical type such as emulsification, spray drying or centrifugal extrusion, and of the chemical type such as ionotropic gelling, liposome entrapment or cyclodextrin complexation. The application of nanotechnology represents the most innovative approach to improve bioavailability and bioactivity of diet-derived phytochemicals [334]. In fact, nanoparticles can help in improving solubility and stability of various phytochemical classes, bringing significant advantages in absorbing phase, as well as limiting or completely avoiding premature degradation of molecules in body and promoting an increase in circulation time. Several examples are reported in scientific literature regarding the application of nanoparticles to improve bioactivity of EGCG (from green tea), resveratrol (found in grape), curcumin (found in turmeric), and quercetin (highly represented in red onions) [334]. However, few information is actually available regarding absorption and metabolism of nanoparticles in the gastrointestinal tract, together with their pharmacokinetics. In general, as described in detail, the main problems encountered in use of these plant extracts at a clinical level are related to richness and complexity of their composition, and this aspect is, in turn, closely linked to the potential risk of toxicity of the extracts as well as their stability [335,336]. Considering that different encapsulation strategies can reduce toxicity further studies are extremely worthwhile to provide targeted delivery systems and to solve problems related to a poor stability. Besides, much more studies are needed to address cost-effectiveness and long-term safety of these delivery systems.

## 8. Conclusions

Viruses are the obligate parasites that are composed of genetic material that can be either in the form of DNA or RNA. The host’s cell replicates viruses, thus harming the host organism with viral infections. Viral infections are the most fatal forms of diseases and some forms of them still cannot be completely treated (e.g., hepatitis and HIV), hence development of life prolonging drugs can be of major importance in human health. Although vaccination policies are the most relevant and effective weapons for the control of viral attacks, however it must be taken into account that specific vaccines are not always available and still today many viral infections, often of an endemic nature are present in the world. In light of this, it is of vital importance to open the horizons of modern medicine to new therapeutic potentials such as the use of principles coming from plants. The possibility of using common plants, used for food purposes, to recover biologically active compounds or plant extracts with relevant biological properties, could represent a challenge of great interest for the future of pharmaceutical sciences. History teaches us that viral infections can be treated by antiviral drugs obtained from traditional sources of medicine as plants. Herbal extracts with phytochemicals can inhibit the process of replication at any of the steps in the viral replication cycle. Plants are the major sources of secondary metabolites which can be used for the synthesis of the antiviral medications along with genetic engineering.

In this perspective, the major threats posed by viral agents and the potential of many phytochemicals to combat their virulence are presented in this review. Specifically, attention is focused on several food plants rich in biologically active compounds capable of fighting the most widespread viruses in the world. Reviewing the main sources of phytochemicals, the various compounds with antiviral activity, their mechanisms of action represent a fundamental starting point for the development of future research and the development of new therapeutic aids. Everything passes through knowledge.

Natural compounds can be explored and used as sources for pharmaceuticals by combining newer methods/aspects of drug discovery. The process of drug discovery involves a series of steps including its identification and isolation from the corresponding plant extract, subsequently followed by purification and, finally, verification by clinical trials. Nowadays, pharmaceutical production is also focused of the biologically active compounds obtained by recombinant processes and metabolic engineering. New frontier with tremendous health implications that already received lots of attention within pharmaceutical sciences, relates to nanomedicines. Nanotechnology aims to develop simple, stable and cost-effective nanoparticles that are useful as drug delivery systems. The development of natural drugs for the near future cannot neglect the modern concept of drug delivery, which is essential to ensure correct bioavailability of the new drug.

Today, especially in the light of recent dramatic events such as the coronavirus pandemic, a critical need for new antiviral agents is emerging. The demand for new therapeutic products with antiviral activity is even higher for the alarming increase in drug resistance and associated problems. For these reasons, systematic continuous exploration of plants and their phytochemicals is recommended for development of new antiviral agents that can save human lives and improve their wellbeing.

## Data Availability

Not applicable.
